# Early Priming Minimizes the Age-Related Immune Compromise of CD8^+^ T Cell Diversity and Function

**DOI:** 10.1371/journal.ppat.1002544

**Published:** 2012-02-23

**Authors:** Sophie A. Valkenburg, Vanessa Venturi, Thurston H. Y. Dang, Nicola L. Bird, Peter C. Doherty, Stephen J. Turner, Miles P. Davenport, Katherine Kedzierska

**Affiliations:** 1 Department of Microbiology and Immunology, University of Melbourne, Parkville, Melbourne, Australia; 2 Computational Biology Group St Jude Children's Research Hospital, Memphis, Tennessee, United States of America; 3 Department of Immunology, St Jude Children's Research Hospital, Memphis, Tennessee, United States of America; 4 Complex Systems in Biology Group, Centre for Vascular Research, University of New South Wales, Kensington, Australia; University of Pennsylvania, United States of America

## Abstract

The elderly are particularly susceptible to influenza A virus infections, with increased occurrence, disease severity and reduced vaccine efficacy attributed to declining immunity. Experimentally, the age-dependent decline in influenza-specific CD8^+^ T cell responsiveness reflects both functional compromise and the emergence of ‘repertoire holes’ arising from the loss of low frequency clonotypes. In this study, we asked whether early priming limits the time-related attrition of immune competence. Though primary responses in aged mice were compromised, animals vaccinated at 6 weeks then challenged >20 months later had T-cell responses that were normal in magnitude. Both functional quality and the persistence of ‘preferred’ TCR clonotypes that expand in a characteristic immunodominance hierarchy were maintained following early priming. Similar to the early priming, vaccination at 22 months followed by challenge retained a response magnitude equivalent to young mice. However, late priming resulted in reduced TCRβ diversity in comparison with vaccination earlier in life. Thus, early priming was critical to maintaining individual and population-wide TCRβ diversity. In summary, early exposure leads to the long-term maintenance of memory T cells and thus preserves optimal, influenza-specific CD8^+^ T-cell responsiveness and protects against the age-related attrition of naïve T-cell precursors. Our study supports development of vaccines that prime CD8^+^ T-cells early in life to elicit the broadest possible spectrum of CD8^+^ T-cell memory and preserve the magnitude, functionality and TCR usage of responding populations. In addition, our study provides the most comprehensive analysis of the aged (primary, secondary primed-early and secondary primed-late) TCR repertoires published to date.

## Author Summary

The elderly population is particularly susceptible to novel infections, especially the annual, seasonal epidemics caused by influenza viruses. Established T cell immunity directed at conserved viral regions provides some protection against influenza infection and promotes more rapid recovery, thus leading to better clinical outcomes. We asked whether priming early in life limits the time-related attrition of immune competence. We found that although influenza-specific T cell responses are compromised in the aged mice, vaccination with influenza early (but not late) in life ‘locks’ optimal T-cell responsiveness, maintains functional quality, persistence of preferred clones and a characteristic T cell hierarchy. Overall, our study supports development of vaccines that prime T cells early in life to elicit the broadest possible spectrum of pre-existing T cell memory and preserve the magnitude, functionality and clonal usage of responding populations for life-long immunity against influenza viruses.

## Introduction

The elderly population is particularly susceptible to novel infections, especially the annual, seasonal epidemics caused by influenza A viruses [1], [2], with increased occurrence, severity of infection and reduced vaccine efficacy being attributed to age-related decline in immune capacity [3]–[6]. The ageing effect on the immune system is considered to be multifactorial, arising from the diminished thymic export of naïve precursors due to thymic involution [7], [8], the impaired recruitment [9], [10] of naïve CD8^+^ T cell precursors and the replicative senescence of memory cells [11]–[14]. Ageing can also be associated with abnormal cellular functions such as distorted cytokine secretion (IL-2, IL-4 and IFN-γ) profiles [15]–[17], decreased granzyme B production [18], [19] and reduced proliferative capacity due to the loss of CD28 expression [20]. Perturbations in the naïve TCR repertoire have also been reported, with abnormal TCR spectratype (CDR3β length) patterns in aged mice reflecting the massive, antigen-independent expansion, of a few clonotypes [21]. Naïve T cell attrition has also been inferred from observed reductions in the diversity of antigen-specific TCR repertoires in aged mice [5], [22].

Previous mouse studies have established that ageing can be associated with diminished CD8^+^ T cell efficacy and delayed influenza virus clearance [23]–[25]. Recent evidence has further shown that the selective loss of primary, influenza-specific CD8^+^ T cell responsiveness in older mice is characterized by a narrowing in the spectrum of TCR usage and is seen predominantly for low frequency populations, with this effect being best characterized for the prominent D^b^NP_366_^+^CD8^+^ T cell set [5], [26]. Overall, the findings so far suggest that the capacity to respond effectively to new influenza infections in aged mice requires the maintenance of a diverse pool of functional peripheral T cells.

As CD8^+^ T cells tend to be specific for peptides derived from more conserved proteins that are internal to the virus, priming effective CD8^+^ T cell memory has obvious potential for countering newly emerged seasonal or pandemic influenza strains. The importance of long-lived, antigen-specific memory CD8^+^ T cells capable of rapid recall following the secondary infection has been well documented for the respiratory viruses in mice [27], [28] and humans [29], [30]. Such long-term maintenance of memory T cells leading to enhanced secondary response forms the basis for vaccination strategies based on priming CD8^+^ T cell memory to promote early virus clearance and decreased morbidity. The question is though, whether such CD8^+^ T cell memory can be effectively recalled in the elderly.

A recent study [6] suggested that infecting mice with LCMV or influenza at an extreme age (18–20 months) leads to defective CD8^+^ T cell memory and diminished recall responses following virus challenge. What happens, though, if CD8^+^ T cell memory is established when the mice are young? The analysis reported here compares the CD8^+^ T cell response profiles for young (<3 months) and aged (22 month) mice, with the latter cohort being first exposed to immunogenic influenza epitopes early or late in life. The results suggest that designing influenza vaccines which promote as broad as possible spectrum of CD8^+^ T cell memory in adolescence could be beneficial, even if such benefit emerges long after the subject has first been given the protective immunogen.

## Results

**Figure 1:**
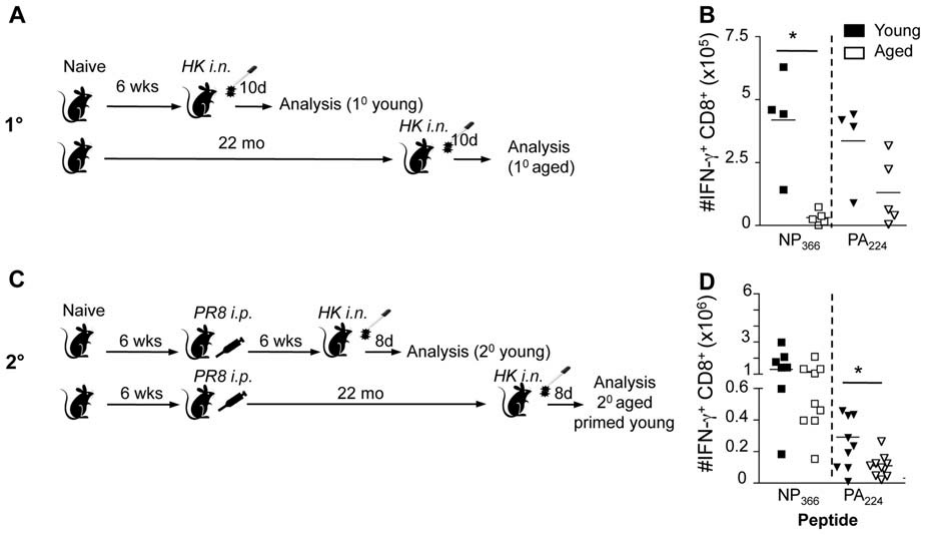
Effect of age and early priming on 1^0^ and 2^0^ CD8^+^ T cell responses. (A) For the primary responses, naïve mice were infected i.n. with 1×10^4^ pfu of the HK (H3N2) influenza A virus either at a young (<3 months; mo) or extreme (22 mo) age. Analysis of CD8^+^ T cell responses was performed on d10 after the primary infection. (B) For the secondary responses of the early-primed mice, animals were primed at <2 mo i.p. with 1.5×10^7^ pfu the PR8 (H1N1) influenza A virus, then challenged 6 weeks (young) or >22 mo (aged) later i.n. with 1×10^4^ pfu of the HK virus. Analysis of CD8^+^ T cell responses was performed on d8 after the secondary infection. (C, D) Numbers of epitope-specific CD8^+^ T cells in the spleens recovered from young (filled symbols) or aged (>22 month, open symbols) B6 mice on d10 (1^0^, C) or d8 (2^o^, D) following primary (1^0^) or secondary (primed young) (2^0^) i.n. infection with the HK (H3N2) influenza A virus. Memory mice had been injected i.p. with the PR8 (H1N1) influenza A virus at <2 mo and were challenged. Lymphocyte populations were stimulated with the NP_366_ or PA_224_ peptides in the presence of Brefeldin A for 5 hrs, then stained with the anti-CD8PerCPCy5.5 mAb, fixed/permeabilised and stained with anti-IFN-γ-FITC mAb. Cytokine (IFN-γ) production was calculated by subtracting background fluorescence for the no-peptide controls, and the numbers of IFN-γ^+^CD8^+^ D^b^NP_366_- and D^b^PA_224_-specifc CD8^+^ T cells were determined from the % cells staining and the total cell counts. Data represent individual mice (symbols) and the mean (line). Experiments were performed at least twice. * = p<0.05.

To validate the previous studies [5], [31] and determine the ageing effect on primary immune responsiveness (Figure 1A) for immunodominant D^b^NP_366_^+^ and D^b^PA_224_^+^ CD8^+^ pools, we infected young (<3 month) and old (>22 month) mice intranasally (i.n.) with 1×10^4^ pfu of an infectious (H3N2, HK) influenza A virus. More importantly, as a main question of the present study, we asked whether any age-related compromise of CD8^+^ T cell function and diversity might be modified by priming early (at 2 months) or late (at 22 months) i.p. with 1.5×10^7^ pfu of the serologically distinct PR8 (H1N1) virus that has the same immunogenic CD8^+^ T cell peptides as HK.

We used the i.p. priming route with the influenza virus as it does not lead to a productive viral replication (similarly to the current i.m. human influenza vaccines), but gives one-stop growth cycle with full protein production. Such non-productive immunisation with the whole virus results in priming of antigen-specific effector T cells and establishment of long-term T cell memory for subsequent challenge (Figure S1), comparable to those observed after the natural (i.n.) influenza infection [32]–[34]. Importantly, the i.p. priming does not elicit the whole cascade of detrimental inflammatory responses in the virally-infected lung [35] and thus avoids double pathology at the site of infection. The i.p. route of influenza priming is equivalent to the current i.m. vaccination approaches used in humans with respect to the non-productive viral immunisation.

### CD8^+^ T cell responsiveness following early versus late infection of aged mice

The comparison of the HK-induced CD8^+^ T cell responses utilized young or old mice that were either immunologically naïve (primary, 1^0^; Figure 1A) or had been primed at 2 months of age with the PR8 virus and challenged 20 months later (secondary, 2^0^; Figure 1B). Immunodominant and subdominant CD8^+^ T cell responses were measured in the spleen (Figure 1CD) by the *ex vivo* IFN-γ ICS assay. Following 1^0^ challenge, the size of the low precursor frequency D^b^NP_366_^+^CD8^+^ set in the spleen (Figure 1C) was markedly diminished in the aged animals relative to the young controls as previously observed [5], [31].

Conversely, any age-related effects on CD8^+^ T cell numbers were not significant for D^b^PA_224_ (Figure 1C). The unaffected D^b^PA_224_^+^CD8^+^ T cell responses are intriguing, as the naïve CD8^+^ T cell frequencies [36] found for D^b^PA_224_–specific T cells in young mice are significantly higher than those detected for D^b^NP_366_ (≥72 versus <40 per individual, respectively), suggesting that a larger naïve CD8^+^ T cell pool size minimizes the extent of age-related attrition and, as a consequence, the effect on primary CD8^+^ T cell response magnitude (Figure 1C).

Reduced magnitude of the immunodominant D^b^NP_366_-specific CD8^+^ T cell response that was detected for the primary, influenza-specific CD8^+^ T cell response in older mice (Figure 1CD), was not sustained following secondary HK challenge of mice that had been primed early with the PR8 virus (Figure 1D). The numbers of D^b^PA_224_CD8^+^ T cells were significantly diminished across combined experiments but, otherwise, the recall responses for memory T cell pools in young or old mice primed at <2 months (at least 20 months previously) were not obviously different, emphasizing the durability of virus-specific CD8^+^ T cell memory [37]. In particular, the overdominance of the D^b^NP_366_-specific set that is characteristic of the secondary response to these viruses [38] was still apparent in the aged mice (Figure 1D).

**Figure 2:**
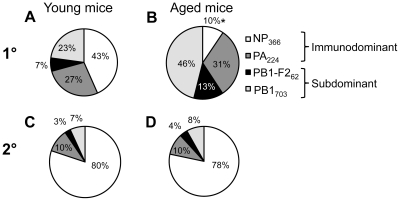
Immunodominance hierarchies in aged mice after 1^0^ infection or 2^0^ challenge of primed-early mice. The relative prevalence of the immunodominant D^b^NP_366_^+^CD8^+^ and D^b^PA_224_^+^CD8^+^ T cell population over the subdominant D^b^PB1_703_^+^CD8^+^ and K^b^PB1-F2_62_^+^CD8^+^ sets. Results are shown for (A, B) 1^0^ and (C, D) 2^0^ HK infection in (A, C) young and (B, D) aged mice. The relative contributions of particular antigen-specific CD8^+^ T cells were analysed based on total cell responses (Figure 1 for D^b^NP_366_^+^CD8^+^ and D^b^PA_224_^+^CD8^+^ and data not shown for D^b^PB1_703_^+^CD8^+^ and K^b^PB1-F2_62_^+^CD8^+^). Data represent the mean proportion of a particular peptide-specific CD8^+^ population. * = p<0.01 shows a difference between young and aged animals. Experimental outline as in Figure 1AB.

The beneficial effect of the early CD8^+^ priming on the immunodominant low-precursor responses like the D^b^NP_366_-specific population following influenza infection at the extreme age was most striking when the relative contributions of particular antigen-specific CD8^+^ T cells were analysed based on total cell numbers (Figure 2, calculations based on Figure 1 for immunodominant D^b^NP_366_^+^CD8^+^ and D^b^PA_224_^+^CD8^+^ pools, and data not shown for subdominant D^b^PB1_703_^+^CD8^+^ and K^b^PB1-F2_62_^+^CD8^+^ populations). In the aged mice, the primary CD8^+^ T cell responses showed a shift in the typical immunodominance hierarchy (Figure 2B), with the contribution of the immunodominant D^b^NP_366_^+^CD8^+^ population being significantly lower in the aged mice (9.4±3.6%) in comparison to young animals (43.4±15%; p<0.01; Figure 2A). The differential immunodominance hierarchy resulted mainly from significantly increased contribution of K^b^PB1_703_^+^CD8^+^ T cells (Figure 2). This led to major modifications in response hierarchy following primary influenza virus infection of aged mice K^b^PB1_703_>D^b^PA_224_>D^b^PB1-F2_62_>D^b^NP_366_, with the comparable profile for young mice being D^b^NP_366_>D^b^PA_224_ = K^b^PB1_703_≫D^b^PB1-F2_62_.

Conversely, recall of CD8^+^ T cells primed at a young age preserved the overall contribution of T cell specificities and retained the immunodominance hierarchy in aged mice primed early at 6 weeks (Figure 2D), reflecting the characteristic immunodominance hierarchy in young controls (Figure 2C). These findings show clearly that priming the CD8^+^ T cell compartment at an early age leads to subsequent preservation of CD8^+^ T cell numbers and immunodominance hierarchies for influenza infection in the elderly.

### Age-related effects on cytokine polyfunctionality and activation status

**Figure 3:**
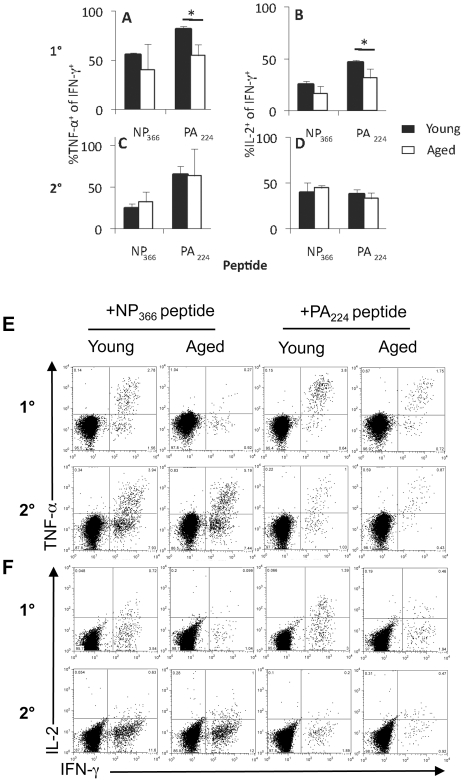
Cytokine polyfunctionality following 1^0^ or 2^0^ challenge. Epitope-specific CD8^+^ T cells generated following 1^0^ (A, B) or 2^0^ (C, D) i.n HK challenge (see legend to Figure 1) of young (black bar) and aged (white bar) mice were assessed for the simultaneous production of IFN-γ, TNF-α (A, C) and IL-2 (B, D) using the ICS assay. The % values (A–F) were compared for spleens from groups of 3–5 mice and representative dot plots are shown (E, F). * = p<0.05. Experimental outline as in Figure 1AB.

**Figure 4:**
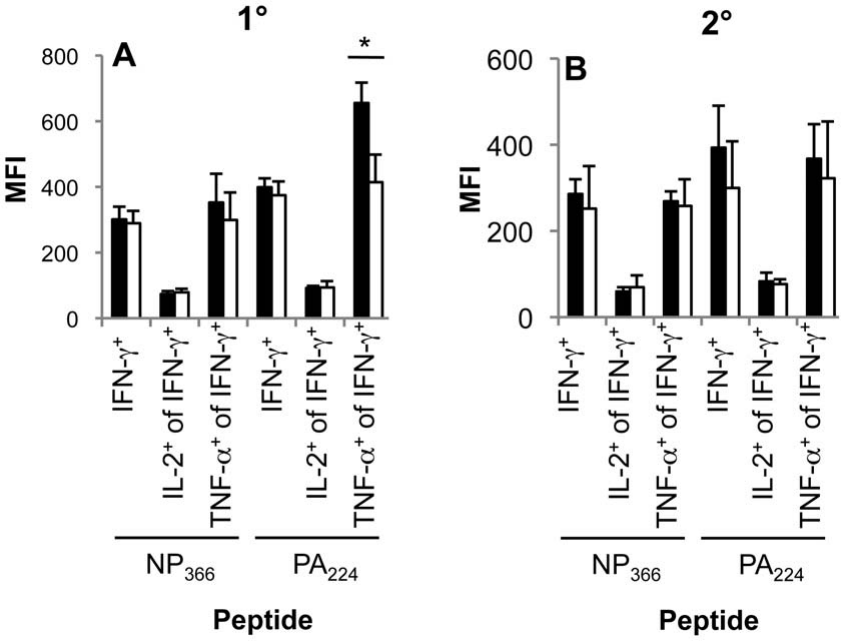
Impaired polyfunctionality of D^b^PA_224_-specific CD8^+^ T cells in the aged mice during primary but not secondary influenza infection. (A) Primary or (B) secondary (primed young) influenza-specific CD8^+^ T cell responses were assessed for simultaneous production of IFN-γ, TNF-α and IL-2 in the spleen of aged (22 months old) and young (6–8 weeks) mice. Compiled data (n = 3–5, mean±SD) are shown for the mean fluorescence intensity (MFI) of IFN-γ, IFN-γ and TNF-α as well as IFN-γ and IL-2 staining. * = p<0.05. Experimental outline as in Figure 1AB.

One measure of CD8^+^ T cell function is the capacity to produce multiple cytokines simultaneously [39] following *in vitro* stimulation with peptide in the standard, 5 h ICS assay. For the primary D^b^PA_224_^+^CD8^+^ T cell population that remained relatively constant in numbers with age (Figure 1C), the frequencies of double (IFN-γ/TNF-α) and triple-producers (IFN-γ/TNF-α/IL-2) were significantly lower in comparison with the young mice (Figure 3AB). Furthermore, taking mean fluorescence intensity (MFI), which represents the intensity and therefore amount of cytokine production, it also seems that the D^b^PA_224_^+^CD8^+^ population tended to produce less TNF-α, though this diminution effect was not apparent for either IFN-γ or IL-2 (Figure 4A). Taking the prevalence and MFI data together (Figure 3 and Figure 4), there appears to be a general decrease in cytokine polyfunctionality for the primary D^b^PA_224_^+^CD8^+^ response.

Conversely, analysis of aged mice primed early showed that functional characteristics appear to be locked-in early and maintained in the long-term for memory T cell populations (Figure 3CDEF).

Can we detect other evidence of enduring functional change? Given that the influenza-specific CD8^+^ T cells generated following primary infection of aged mice were either of suboptimal functional quality (D^b^PA_224_^+^CD8^+^; Figure 3, Figure 4) or reduced in number (D^b^NP_366_^+^CD8^+^; Figure 1), the further question was whether there was any effect on cell surface activation phenotype [34], [40]–[42]. Comparison of phenotypic markers associated with activation, trafficking and memory potential: CD62L vs. IL-7Rα (CD127), CD27 vs. CD43, and IL-7Rα vs. KLRG-1 for the D^b^PA_224_^+^CD8^+^ and D^b^NP_366_^+^CD8^+^ sets (Figure S2) showed that, with the exception of a decrease in the relative prevalence of the less activated CD27^lo^CD43^lo^CD8^+^ D^b^PA_224_^+^ cells in the older mice (Figure S2AD), there were no significant differences in phenotype with age.

### Aged memory T cells have a young-type TCRβ usage profile

**Figure 5:**
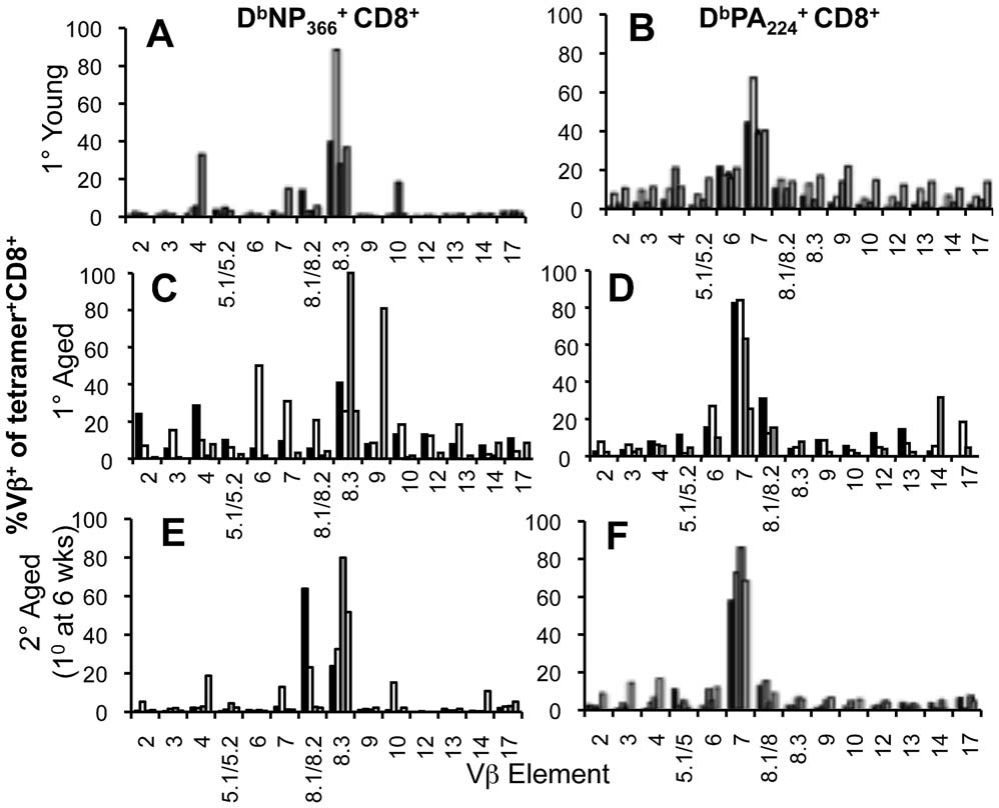
Profiles of Vβ usage for tetramer^+^ CD8^+^ T cells. Profiles of TCR Vβ usage are shown for d10 (1^0^, A–D) or d8 (2^0^, early priming EF) CD8^+^ T cells from young (AB) or aged (C–F) mice. The splenocytes were stained with D^b^NP_366_ (ACE) and D^b^PA_224_ (BDF) PE tetramers, anti-CD8-APC and a panel of anti-Vβ mAbs conjugated with FITC. Results represent individual mice of 4 per group. Experimental outline as in Figure 1AB.

Previous studies have found a significant skewing in TCR Vβ usage (mAb staining) and CDR3β length (spectratyping) for CD8^+^ T cell responses developed from naïve and memory populations by the infection of aged versus young mice [13], [21], [43]. Thus, we looked more closely at the expansion and maintenance of responding T cell clonotypes [44], [45]. As our earlier analysis of influenza-specific CD8^+^ TCR clonotype diversity has focused on the prominent Vβ8.3^+^D^b^NP_366_^+^ [44], [46] and Vβ7^+^D^b^PA_224_^+^ sets [47], we first assessed the Vβ mAb-staining profiles to determine whether these characteristic TCRs were also selected following primary or secondary challenge of aged mice. Indeed for both D^b^NP_366_^+^ CD8^+^ and D^b^PA_224_^+^CD8^+^ T cell responses, the characteristic Vβ8.3 and Vβ7 usage was observed (Figure 5), though additional Vβ6, Vβ7 and Vβ9 biases were variously detected in individual, older mice for the primary D^b^NP_366_^+^CD8^+^ population (Figure 5C), possibly due to the recruitment of low frequency alternate D^b^NP_366_-specific CD8^+^ T cells. Despite the presence of a prominent Vβ8.1/8.2^+^D^b^NP_366_^+^ set in one of the early-primed, secondarily-challenged at 22 month mice, the bias was generally to Vβ8.3 suggesting that the characteristic D^b^NP_366_^+^CD8^+^ TCRβ usage profile is retained in the persistent memory population. The D^b^PA_224_^+^ set was characterised across groups by Vβ7 TCR usage (Figure 5BDF), which was more consistent than the D^b^NP_366_^+^Vβ8.3 usage, possibly reflecting the higher number of precursors with Vβ7 surviving within the 22 month old mice.

### Priming at an extreme age does not impair the recall response magnitude

**Figure 6:**
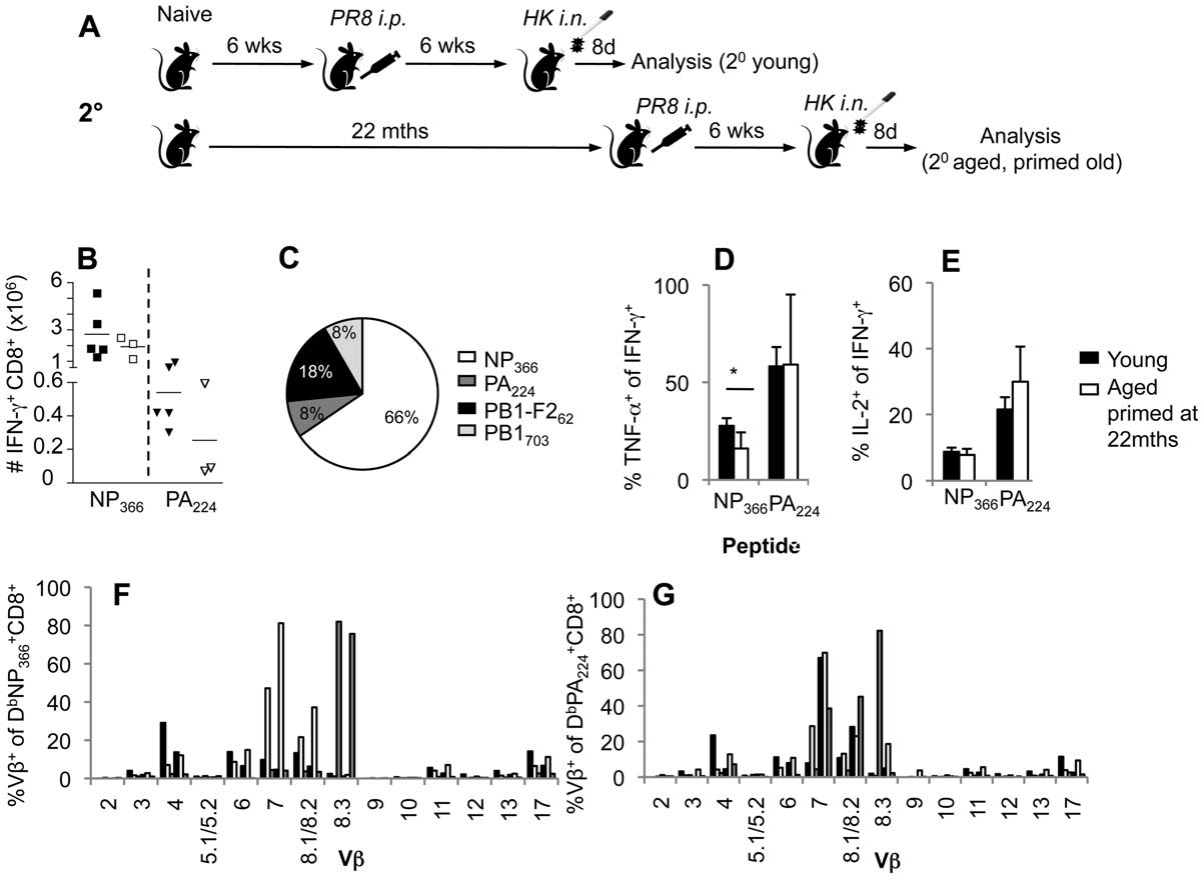
Priming at an extreme age leads to normal secondary influenza-specific CD8^+^ T cell responses. (A) For the secondary responses of the old-primed mice, naïve B6 mice were i.p. primed with 1.5×10^7^ pfu of the PR8 virus either at 6 weeks of age (young mice) or at 22 months (primed late aged mice), followed by a secondary i.n. challenge with 1×10^4^ pfu of the HK influenza strain 6 weeks later. (B) The magnitude of CD8^+^ T cell responses in the spleen at the peak (d8) of secondary phase following influenza virus infection are shown for young (6–8 weeks) and aged (22 months old) B6 mice. Immunodominant D^b^NP_366_^+^ and D^b^PA_224_^+^ influenza-specific CD8^+^ T cell responses were assessed by IFN-γ production in an *ex vivo* ICS assay. (C, D) Polyfunctionality of influenza-specific CD8^+^ T cell responses was assessed by simultaneous production of IFN-γ, TNF-α and IL-2 in the spleen and of young and aged mice. (E) The contribution of immunodominant D^b^NP_366_^+^CD8^+^ and D^b^PA_224_^+^CD8^+^ T cell responses in comparison to subdominant D^b^PB1_703_^+^CD8^+^ and K^b^PB1-F2_62_^+^CD8^+^ sets was calculated based on the proportions of IFN-γ^+^CD8^+^ populations depicted in (B for D^b^NP_366_^+^CD8^+^ and D^b^PA_224_^+^CD8^+^ and data not shown for D^b^PB1_703_^+^CD8^+^ and K^b^PB1-F2_62_^+^CD8^+^). TCR Vβ usage for the (F) D^b^NP_366_ and (G) D^b^PA_224_ CD8^+^ sets in the spleen of recall responses of mice primed late. TCR Vβ results represent individual mice of 3 per group. * = p<0.05.

Since priming at a young age led to the typical magnitude and quality of influenza-specific CD8^+^ T cell responses following viral infection in the aged mice, we asked whether priming the mice via a non-replicative route (i.p. priming with 1.5×10^7^ pfu of PR8) at extreme age (22 months) would be also beneficial for the subsequent influenza virus infection. Since the reduced primary D^b^NP_366_^+^CD8^+^ T cell responses in aged mice has been attributed to the lower naïve precursors in young mice [5], this experiment would determine whether old naive mice could be primed at an extreme age (at 22 months) and subsequently challenged i.n. with 1×10^4^ pfu of the HK influenza strain (at ∼24 months; Figure 6A) to mount an effective recall response after the attrition had occurred. Surprisingly, despite the reduced primary D^b^NP_366_^+^CD8^+^ T cell responses (Figure 1C) and lower magnitude of secondary D^b^PA_224_^+^CD8^+^ sets (Figure 1E) in the spleens of aged animals, the recall of influenza-specific CD8^+^ T cells was robust and equivalent in magnitude to the young controls (Figure 6). The numbers of both immunodominant D^b^NP_366_^+^CD8^+^ and D^b^PA_224_^+^CD8^+^ populations were normal (Figure 6B). This resulted in the maintained contribution of each of the T cell specificities to influenza-specific responses (Figure 6E). Conversely, the polyfunctionality of those secondary CD8^+^ T cell populations in mice primed at the extreme age did not always resemble effectiveness of influenza-specific CD8^+^ T cells recruited in young individuals (Figure 6C). Perturbations in the TCR usage with extreme age were evident macroscopically in the TCR Vβ usage for D^b^PA_224_^+^CD8^+^ (Figure 6G) and especially the D^b^NP_366_^+^CD8^+^ (Figure 6F) responses, with the usage of alternate Vβ8.1/8.2 for D^b^PA_224_^+^CD8^+^, and Vβ7 and Vβ8.1/8.2 for D^b^NP_366_^+^CD8^+^ populations. The characteristic Vβ8.3 usage for D^b^NP_366_^+^CD8^+^ was only dominant in 1 of 4 mice (Figure 6F), reflecting narrowing of the naïve D^b^NP_366_^+^CD8^+^ set with extreme age that initially limited the primary response (Figure 1C) and/or the clonal expansions characteristic for the aged animals as previously reported [13], [14].

### Early but not late priming preserves TCRβ usage of ‘preferred’ clonotypes in the aged mice

**Figure 7:**
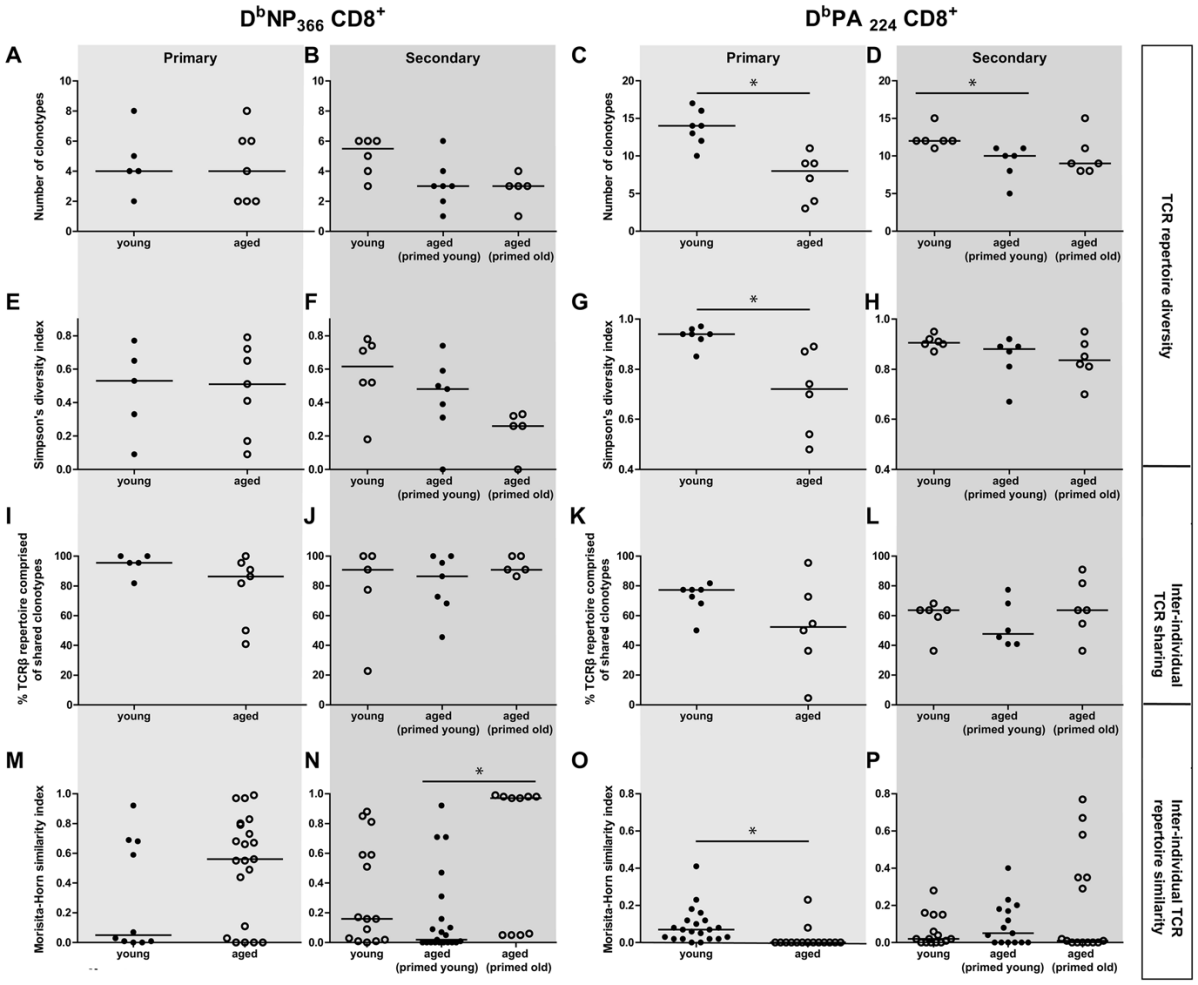
Comparison of TCRβ diversity, and inter-individual sharing and similarity for the D^b^NP_366_^+^Vβ8.3^+^CD8^+^ and D^b^PA_224_^+^Vβ7^+^CD8^+^ repertoires. Shown are the relative measures of TCR**β** repertoire diversity, (A–D) the number of different clonotypes and (E–H) Simpson's diversity index, and (I–L) % of repertoire comprised of shared clonotypes, and (M–P) inter-individual TCR repertoire similarity, as measured by the Morisita-Horn similarity index. The Simpson's diversity and Morisita-Horn similarity indices account for the clonal dominance hierarchy among the different clonotypes and vary between 0 (minimum diversity/similarity) and 1 (maximum diversity/similarity). Each of the diversity, inter-individual sharing and similarity measures were estimated for a standard sample size of 22 TCR sequences per individual mouse repertoire. The repertoire diversities were calculated for each mouse per age/priming group for primary (A, E) and secondary (B, F) D^b^NP_366_^+^Vβ8.3^+^CD8^+^ TCR repertoires and for primary (C, G) and secondary (D, H) D^b^PA_224_^+^Vβ7^+^CD8^+^ TCR repertoires. The repertoire similarities were assessed between pairs of primary (M) and secondary (N) D^b^NP_366_^+^Vβ8.3^+^CD8^+^ TCR repertoires and between pairs of primary (O) and secondary (P) D^b^PA_224_^+^Vβ7^+^CD8^+^ TCR repertoires within the same age/priming group. To evaluate TCR sharing, clonotypes were first defined as shared or non-shared across all D^b^NP_366_-specific or D^b^PA_224_-specific TCRβ repertoires. The proportions of the 22 TCRβ sequences per D^b^NP_366_^+^Vβ8.3^+^CD8^+^ TCR repertoire (I, J) or D^b^PA_224_^+^Vβ7^+^CD8^+^ TCR repertoire (K, L) that were comprised of shared clonotypes were then estimated. A Mann-Whitney test was used to compare between young and aged mice for the primary responses and between young mice, aged mice primed young and aged mice primed old for the secondary responses. For the comparison between age/priming groups for the secondary responses, the statistical significance for each pairwise comparison was determined at p<0.0167 (*), using Bonferroni correction for multiple pairwise comparisons.

**Figure 8:**
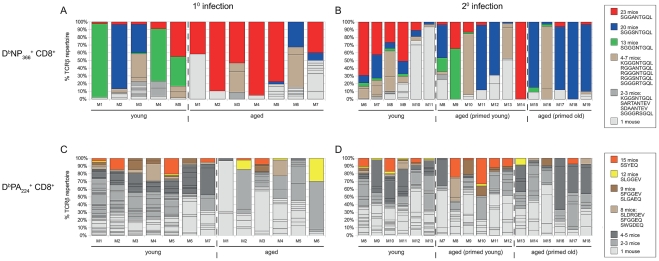
Comparison between aged and young mice of the dominance of shared D^b^NP_366_^+^CD8^+^ Vβ8.3^+^ and D^b^PA_224_^+^CD8^+^ Vβ7^+^ TCR clonotypes during primary and secondary (primed-young and primed-old) infections. Shown are the percentages of the D^b^NP_366_^+^CD8^+^Vβ8.3^+^ (A, B) and D^b^PA_224_^+^CD8^+^Vβ7^+^ (C, D) TCR repertoires per mouse that are comprised of aa clonotypes shared between a particular number of mice (indicated by colour-coding) during primary (A, C) and secondary (B, D) infections. The number of mice sharing a TCR clonotype was determined across young and aged mice and both primary and secondary challenges. For example, the public D^b^NP_366_^+^CD8^+^Vβ8.3^+^ TCR clonotype SGGANTGQL (red) was observed in 23 out of 30 mice (A, B). This clonotype contributed to 95% of the primary D^b^NP_366_^+^CD8^+^Vβ8.3^+^ TCR repertoire of aged mouse M4 (A). There were five D^b^NP_366_^+^CD8^+^Vβ8.3^+^ TCR clonotypes that were each observed in a number of mice ranging between 4 and 7 mice (beige). Three of these five clonotypes contributed to the D^b^NP_366_^+^CD8^+^Vβ8.3^+^ TCR repertoire responding to secondary infection in young mouse M7 (B, as indicated the three beige segments). Multiple unshared clonotypes (light grey), which were observed in only one mouse, contributed to the TCR repertoires of many of the mice (as indicated by multiple light grey segments per column).

A substantial body of work from previous studies has defined the young B6 CDR3β TCR usage at high resolution [44], [47], therefore using these data sets from young mice we were able to compare the spectrum of clonotype prevalence in aged mice using single-cell RT-PCR and sequencing of the CDR3β region to determine the spectrum of TCRβ diversity. Analysis of 1489 CDR3β sequences for primary and secondary (primed-young and primed-old) responses from the 22 month old mice (Tables 1 and 2) showed that the dominant Jβ regions and CDR3β loop lengths in the aged animals (Tables S1, S2, S3, S4, S5, S6) were comparable to those found early in life (Figures 7 and 8 for comparison with young animals). However, more inter-individual variation in the primary responses was observed in the older group (Figure S3). While >83% of each of the TCRβ repertoires involved in the primary responses to D^b^NP_366_ in young mice utilized Jβ2.2 and a CDR3 length of 9 amino acids (aa), this profile was substantially diminished to <57% of the TCRβ repertoire for 2/7 aged mice. Similarly, Jβ1.1, Jβ1.5, and Jβ2.6 collectively dominated the primary D^b^PA_224_^+^CD8^+^ responses for 7/7 young mice, while Jβ2.1 and Jβ2.3 emerged strongly (>55% each) for 2 of the older mice. While the primary D^b^PA_224_^+^CD8^+^ repertoires in individual young mice mostly featured diverse CDR3 lengths of 5, 6, and 7 aa, >94% of the primary D^b^PA_224_^+^CD8^+^ T cell repertoires in two of the aged mice could be attributed to one particular CDR3 length (i.e. 6 aa in one mouse and 7 aa in the other mouse).

**Table 1:** CDR3β TCR repertoire of D^b^NP_366_^+^Vβ8.3^+^CD8^+^ T cells at the acute phase of primary and secondary influenza virus infection of young and aged mice.

	Primary		Secondary		
D^b^NP_366_^+^Vβ8.3^+^CD8^+^	Young	Aged	Young	Aged primed young	Aged primed old
Mice analysed	5	7	6	7	5
TCRs sequenced	287	284	383	358	153
Different clonotypes (aa)	24	29	22	15	9
Different clonotypes (nt)	37	37	41	29	16
Clonotypes per mouse (aa)	7.0±5.1	5.1±3.5	6.5±2.3	3.6±1.8	3.2±1.5
Clonotypes per mouse (nt)	8.6±5.6	5.6±3.8	8.8±4.2	4.7±1.4	4.4±1.1

a Predominant when found in more than 15% of mice.

**Table 2:** CDR3β TCR repertoire of D^b^PA_224_^+^Vβ7^+^CD8^+^ T cells at the acute phase of primary and secondary influenza virus infection of young and aged mice.

	Primary		Secondary		
D^b^PA_224_^+^Vβ7^+^CD8^+^	Young	Aged	Young	Aged primed young	Aged primed old
Mice analysed	7	6	6	6	6
TCRs sequenced	373	277	347	249	168
Different clonotypes (aa)	115	55	105	66	54
Different clonotypes (nt)	150	58	128	79	55
Clonotypes per mouse (aa)	23.9±6.8	10.0±4.8	21.8±3.8	13.3±3.7	11.0±2.4
Clonotypes per mouse (nt)	26.0±8.1	10.2±5.0	23.5±4.0	13.8±4.2	11.0±2.4

b Repeated (shared) when sequence found in more than 80% of mice.

Age-associated changes in TCRβ repertoire usage were investigated for the D^b^NP_366_^+^CD8^+^ and D^b^PA_224_^+^CD8^+^ populations by sequencing individual CDR3β TCR signatures (Tables 1 and 2, Tables S1, S2, S3, S4, S5, S6) and the extent of TCRβ repertoire diversity was then assessed using both the number of different aa-defined clonotypes and Simpson's diversity index, which accounts for the clonal dominance hierarchy. These measures of diversity were estimated for a standard 22 TCRβ sequences per epitope per mouse to adjust for differences in total number of sequences obtained per mouse [48]. The primary D^b^PA_224_^+^CD8^+^ TCRâ repertoires were found to be significantly less diverse in aged versus young mice, with a lower number of clonotypes per individual (median: 8 vs. 14, p = 0.005; Figure 7C) and a decreased Simpson's diversity index (median: 0.72 vs 0.94, p = 0.007; Figure 7G), despite there being no significant change in the D^b^PA_224_-specific CD8^+^ T cell response magnitude (Figure 1A). Some age-related contraction in the number of different D^b^PA_224_^+^CD8^+^ TCRâ clonotypes was also found following secondary infection (early priming) (median: 10 vs. 12, p = 0.007; Figure 7D), though the difference was not as large as in the primary response, largely due to the increased median diversity for the recall response in older mice. Interestingly, when mice were primed at 22 months of age and then challenged (primed old), similar results were obtained as early priming, however there appeared to be substantial increase in the similarity between some pairs of mice (Figure 7P). Surprisingly, the reduced diversity seen in the D^b^PA_224_^+^ CD8^+^ primary response (Figure 7CG), from which the late priming response is derived, was not carried over to the primed-old recall TCRβ repertoire (Figure 7DH). This suggests that priming plays a positive role in preserving a broader spectrum of clonotype availability within the inherently diverse D^b^PA_224_^+^CD8^+^ T cell repertoire, due to enhanced response magnitude.

In contrast, despite the greatly diminished magnitude of the primary D^b^NP_366_^+^CD8^+^ T cell response in older mice (Figure 1A), the extent of TCRβ repertoire diversity analysed at the aa level was not significantly different for young and old mice (Figure 7AE, Table 1, Table S1). The public D^b^NP_366_^+^Vβ8.3 clonotypes can be encoded by up to 10 different nucleotide (n.t.) sequences each, with as many as 4 distinct n.t.-defined variants being present in an individual young mouse [44]. Following primary exposure of aged animals or when mice were primed late, the three main public Vβ8.3^+^ D^b^NP_366_^+^CD8^+^ clonotypes: SGGANTGQL, SGGGNTGQL, SGGSNTGQL [44] were encoded by a total of 10 and 9 distinct n.t. sequences respectively (Tables S1 and S5), in contrast to the 16 different clonotypes detected in the secondary-infected (primed early), aged mice (Table S3). As a consequence, priming early or late prior to challenge preserved a mean of 2.9±1.1 and 3.0±0.7 n.t.-defined public clonotypes in comparison to the 1.7±1.1 public n.t. sequences detected following infection of old, naïve mice. Such reduced availability of n.t.-defined public sequences in the primary aged mice resulted in a loss of one of the major public clonotypes SGGGNTGQL (Figure 8A) in all 7 animals tested following primary virus challenge (Table S1). This was associated in turn with a markedly greater contribution of the SGGANTGQL clonotypes (57% versus 23%) in primarily-infected aged animals in comparison to those that were secondary challenged (Figure 8B). It is interesting to note that previously the SGGANTGQL clonotype has been associated with low pMHC avidity [49]. Thus, although the D^b^NP_366_^+^CD8^+^ repertoire is dominated by public TCRs encoded by multiple distinct n.t. sequences, due to codon redundancy the selective, age-related exclusion of one n.t.-defined clonotype does not necessarily equate to the disappearance of any given aa clonotype from the naïve pool. However, it is still possible that the prominent TCR signatures (like SGGGNTGQL) can be lost or significantly decreased with ageing.

Significantly higher inter-individual similarity of D^b^NP_366_TCRβ repertoires was seen in the recall response of aged mice that were primed old compared with aged mice primed young (Figure 7N). The proportion of individual mouse TCRβ repertoires comprised of shared clonotypes was consistently high across age and priming groups (Figure 7IJ). Furthermore, there was higher inter-individual similarity during the secondary D^b^NP_366_^+^CD8^+^ responses in aged mice primed old (Figure 7N) was largely due to the SGGSNTGQL clonotype that was dominant in 4/5 mice, and therefore dominated the primed aged secondary response (Figure 8B, Table S5). The lesser prevalence and dominance of this SGGSNTGQL clonotype in the aged primary response (Figure 8A, Table S1) could be related to the avidity of individual clonotypes recruited during recall and preferential homeostatic proliferation, which is reminiscent of the lower avidity SGGANTGQL clonotype dominating the primary aged response above. Overall, there was a trend towards lower TCR diversity in the D^b^NP_366_^+^CD8^+^ response to secondary infection in aged mice, regardless of age of priming, compared with young mice. However, due to the extreme dominance of SGGSNTGQL (Figure 8B), and the significantly greater inter-individual similarity (Figure 7N) in aged mice primed late versus early, the timing of priming has a narrowing effect on the population-wide Vβ8.3^+^ D^b^NP_366_^+^CD8^+^ TCRβ repertoire. Thus, encountering an immunogenic epitope leads to a relative preservation of TCRβ diversity at the n.t. level (the ‘actual’ clonotypes), even if repertoire diversity at the aa level appears unchanged. Priming also prevents the attrition of dominant public TCRs with age and mediates their recruitment into the CD8^+^ T cell effector pool in the elderly.

The results of the present study also confirm our previous longitudinal analysis of D^b^NP_366_^+^CD8^+^ responses [44] and differential clonotype hierarchy usage in the primary young and secondary young mice (Figures 8A and 8B). While SGGGNTGQL is a preferential clonotype after the i.p. priming (as well as after the primary i.n. infection), the hierarchy changes after re-challenge, with SGGANTGQL and SGGSNTGQL clonotypes dominating the secondary response.

## Discussion

The present analysis establishes the importance of priming the CD8^+^ T cell compartment early in life in order to preserve CD8^+^ T cell numbers, functional quality and preferential profiles of TCR usage for influenza-specific CD8^+^ effector T cell responses in the elderly. In contrast, primary CD8^+^ T cell responses in aged animals tended to show alterations in the typical CD8^+^ T cell immunodominance hierarchy, with T cell responses to some epitopes being reduced in magnitude, a decrease in the capacity to make multiple cytokines, and changes in the extent of TCRβ repertoire diversity as a consequence of the diminished availability of naïve clonotypes. These effects were minimal for the recall responses generated from memory T cell populations that were generated early, and then recalled by virus challenge more than 18 months later. Overall, the results emphasize both the durability and constancy of immune memory.

The response hierarchy following primary influenza virus infection of aged mice was K^b^PB1_703_>D^b^PA_224_>D^b^PB1-F2_62_>D^b^NP_366_, with the comparable profile for young mice being D^b^NP_366_>D^b^PA_224_ = K^b^PB1_703_≫PB1-F2_62_. Typically subdominant epitopes accounted for 59% of the response in aged naïve mice challenged with virus compared with a 34% (Figure 2A) contribution in the young. Thus, immunodominance hierarchies may be relative to age, an idea that is clearly more relevant to the situation in long-lived humans than in mice. In contrast, the typical hierarchy [36] was maintained for both young and old mice that were primed early, with a relative contribution by subdominant epitopes of 10% and 12% (Figure 2D) respectively. Whereas when mice were primed at an extreme age subdominant epitopes contributed 26% of the anti-influenza CD8^+^ T cell response and, therefore, the immundodominance hierarchy was perturbed (Figure 6E), to a lesser extent than the primary response in aged mice.

The difference in naïve precursor frequency for the D^b^NP_366_^+^CD8^+^ and D^b^PA_224_^+^CD8^+^ T cell sets is only two-fold (36 vs 79 naïve precursors, respectively) [36], yet any age-related diminution in magnitude for the primary response to D^b^PA_224_ was less apparent, suggesting that expanding CD8^+^ T cell precursors prevalence by an estimate of 2–4 fold may protect immune capacity in the long term. As all the naïve, endogenous and non-transgenic D^b^NP_366_^+^CD8^+^, and D^b^PA_224_^+^CD8^+^ T cells are recruited into the primary immune response [36], there would be no naïve precursors left to mount a primary CD8^+^ T cell responses after re-challenge for these three sets of influenza-specific CD8^+^ T cell populations, unless new precursors had emerged subsequently from the thymus.

With age, the relative loss in magnitude for the normally prominent D^b^NP_366_-specific response can be most likely attributed to the loss of naïve precursors with time as previously suggested [5]. Despite multiple attempts to repeat the naïve CD8^+^ T cell analysis for aged (22 mo) B6 mice, we were unable to recover viable tetramer^+^CD8^+^ populations (data not shown) following the application of the rigorous magnetic separation procedure that is required to recover very small numbers of antigen-specific cells from the total, peripheral CD8^+^ T cell pool [36], [50] in the aged mice comparing to normal precursor frequencies in the young controls. This could reflect diminished structural integrity due, for instance, to senescence-associated changes in membrane lipids [51]. Thus, at this time we were unable to compare naïve influenza-specific CD8^+^ T cell precursor frequencies of aged mice to established precursor frequencies in the young controls, but rather infer results from the immunodominance hierarchy of the aged primary responses.

The comparable sizes and immunodominance hierarchies of influenza-specific CD8^+^ T cell responses in young and elderly following recall reflects the stability of long term-memory pools, which has also been evidenced by earlier data showing stable memory numbers for both D^b^NP_366_^+^CD8^+^ and D^b^PA_224_^+^CD8^+^ T cells until at least d575 after primary infection [32]. Together with the present analysis, evidence for the preservation of Vaccinia virus-specific memory populations in humans primed more than 20 years previously [52] reinforces the view that early antigen encounter minimizes the attrition of CD8^+^ T cell responses in the elderly. Furthermore, analysis of the 2009 H1N1 (swine-origin influenza) response in human populations showed that this newly emerged pandemic virus shared immunogenic peptides with the catastrophic 1918 H1N1 strain [53], emphasizing the likely value of establishing effective CD8^+^ T cell memory to all known influenza epitopes.

Early priming of the CD8^+^ T cell compartment also preserves CD8^+^ T cell functionality in the very long term. In contrast to the suboptimal peptide-induced, polyfunctional cytokine profiles expressed by CD8^+^ T cells generated from naïve CD8^+^ T cells in aged animals, the recall of influenza-specific CD8^+^ T cell memory in the elderly is associated with functional profiles comparable to those found in the young. Since polyfunctionality (simultaneous IFN-γ, TNF-α and IL-2 production) of CD8^+^ T cells is thought to correlate with protective efficacy [54], [55], [56], establishing optimal cytokine profiles early may provide a clear advantage for virus-specific CD8^+^ T cell responses in the elderly.

Ageing is often associated with the attrition of the peripheral TCR repertoire, reflecting the loss of some T cell clonotypes and the large expansion of others [5], [14], [21]. Our study provides the most comprehensive analysis of the aged (primary, secondary primed-early and secondary primed-late) TCR repertoire published to date. The present, unbiased single-cell RT-PCR analysis of CDR3β usage in the elderly showed a diminished number of clonotypes during the aged primary D^b^PA_224_^+^CD8^+^ responses when compared with the normal profiles for young individuals [44], [47], [57]. As naïve D^b^NP_366_^+^CD8^+^ and D^b^PA_224_^+^CD8^+^ T precursors are efficiently recruited into the primary immune response [36], this primary repertoire analysis can be considered to reflect the loss of a substantial proportion of naïve TCRs with ageing. Whilst a previous study [42] suggested that age-related clonal TCR attrition is more prevalent for the low precursor frequency D^b^NP_366_^+^CD8^+^ repertoire, we found a greater reduction in the numbers of D^b^PA_224_^+^CD8^+^ (down 60.8%) versus D^b^NP_366_^+^CD8^+^ (down 34.9%)-specific nucleotide clonotypes per mouse recovered following primary infection of older mice (Tables 1 and 2). This is likely to reflect that there are a greater variety of n.t. types encoding public D^b^NP_366_-specific aa clonotypes across all mice than for D^b^PA_224_^+^-specific aa clonotypes, which potentially makes D^b^PA_224_^+^CD8^+^ aa-defined clonotypes more vulnerable to total clonotype loss and thus reduced diversity.

The public, aa-defined D^b^NP_366_^+^CD8^+^ CDR3β clonotypes can be encoded by up to 10 different n.t. sequences [44], meaning that the loss of one n.t.-defined public TCR may not necessary result in the elimination of that particular CDR3β aa sequence. Thus, it is not surprising that the D^b^NP_366_^+^CD8^+^ CDR3β clonotypes in the aged mice following primary infection are encoded by a limited number of n.t. sequences (1.7±1.1 per mouse) inferring a loss of D^b^NP_366_-specific CD8^+^ T cells. This was associated with the decreased contribution of two main public clonotypes (SGGGNTGQL and SGGSNTGQL) and the increased prominence of one public clonotype (SGGANTGQL) in aged mice following primary influenza virus challenge. Similar epitope-specific TCRβ repertoire homogenisation across a population of aged mice has been recently observed for CD8^+^ T cell responses to HSV-1 [22]. As SGGANTGQL is of lower pMHC avidity [49], the dominance of this clonotype in the aged repertoire may be one reason for the lower functional quality of D^b^NP_366_^+^CD8^+^ T cell responses in the elderly.

The real advantage of priming CD8^+^ T cell responses early in life is reinforced by the demonstration that n.t.-defined clonotype diversity is preserved for the public D^b^NP_366_^+^ CD8^+^ T cell response, resulting in more equal contribution of the 3 main public clonotypes (SGGANTGQL, SGGGNTGQL and SGGSNTGQL), which was not seen when mice were primed later in life (where SGGSNTGQL alone dominated). Similarly, the secondary D^b^PA_224_^+^CD8^+^ response in aged mice is slightly more diverse than that generated following primary virus challenge. Thus, early priming of the CD8^+^ T cell compartment induces a more diverse, aged repertoire by promoting the survival of public D^b^NP_366_^+^CD8^+^ clonotypes. This may in turn reflect the selection of “best-fit” TCRs. Maintaining TCR repertoire diversity can enhance the efficacy of CD8^+^ T cell-mediated immunity [58], diminish the likelihood that mutated pathogens ‘escape’ immune recognition [59] and lead to more cross-reactive CD8^+^ T cell responses [53], [60]. Preserving a greater breadth of responding TCRs is thus likely to be favorable for the elderly population. Taken together, our study supports the evolution of vaccine strategies to prime CD8^+^ T cells early in life in order to preserve the magnitude, functionality, TCR repertoire diversity and preferential TCR usage of responding populations.

## Materials and Methods

### Ethics statement

All animal experimentation was conducted following the Australian National Health and Medical Research Council Code of Practice for the Care and Use of Animals for Scientific Purposes guidelines for housing and care of laboratory animals and performed in accordance with Institutional regulations after pertinent review and approval by the University of Melbourne Animal Ethics Experimentation Committee in Melbourne.

### Mice and influenza virus infection

Female C57BL/6J (B6, H2^b^) mice were bred and housed under specific pathogen free (SPF) conditions at the Department of Microbiology and Immunology, University of Melbourne. Primary responses: For generation of acute primary influenza CD8^+^ T cell responses, mice were lightly anaesthetised by inhalation of methoxyflurane and infected intranasally (i.n.) with 1×10^4^ plaque forming units (pfu) of H3N2 (HK) influenza A viruses in 30 µµl of PBS. Young mice were infected at 6–8 weeks, while aged mice were infected at 22 months of age. Secondary responses: To study the effects of *early* priming on aged CD8^+^ T cell responses, mice were first primed intraperitoneally (i.p.) at 6 weeks of age with 1.5×10^7^ pfu of H1N1 PR8 influenza A virus and subsequently challenged with the serologically distinct H3N2 HK virus at extreme age of 22 months (6 weeks->22 months; primed young->challenged old). To study the effects of *late* priming on aged CD8^+^ T cell responses, mice were first primed i.p. with PR8 at 22 months and challenged 6 weeks later with HK (22 months ->23.5 months; primed old->challenged old). Control young animals were primed at 6 weeks, then challenged at 12 weeks of age (6 weeks->12 weeks; primed young->challenged young). The aged cohort of mice were held for up to 24 months in SPF conditions, monitored for signs of infection, weight loss and spontaneous tumor growth.

### Tissue sampling and cell preparation

Spleens were recovered from mice at acute phases of the primary and secondary infections (day (d) 10 and d8, respectively). Spleens were depleted of B cells by incubation on αIgG/IgM coated plates (Jackson ImmunoResearch Labs) for 45 mins at 37°C, and unbound cells harvested.

### Tetramer and phenotypic staining of CD8^+^ T cells

Enriched lymphocytes from the spleen were stained with D^b^NP_366_ and D^b^PA_224_ tetramers conjugated to Strepavidin-APC or -PE (Invitrogen) at optimal staining concentrations for 1 hr at room temperature. Cells were then washed twice in FACS buffer (PBS with 1%BSA/0.02% sodium azide) and stained with 1 µg/ml CD8-PerCP Cy5.5 (all BD Biosciences unless stated) plus either: 5 µg/ml CD27-PE and 5 µg/ml CD43-FITC (activation associated glycoform: clone 1B11, eBiosciences) or 5 µg/ml CD62L-FITC and 5 µg/ml CD127-PE (IL-7Rα chain), or 5 µg/ml KLRG1-FITC (Abcam) and 5 µg/ml CD127-PE. For Vβ usage analysis, tetramer-stained cells were incubated a panel of FITC conjugated anti-Vβ mAbs (2, 3, 4, 5.1/5.2, 6, 7, 8.1/8.2, 8.3, 9, 10, 12, 13, 14 and 17) [61] at 5 µg/ml, and 1 µg/ml anti-CD8-PerCPCy5.5. Cells were stained for 30 mins on ice, washed twice and analyzed by flow cytometry using a FACS Calibur (BD Biosciences) and Flowjo software (Treestar).

### Peptide stimulation and intracellular cytokine staining

Splenocytes were stimulated with 1 µM NP_366_ or PA_224_ peptides (AusPep) for 5 hrs at 37°C, 5% CO_2_ in the presence of 1 µg/ml Golgi-Plug (BD Biosciences) and 10 U/ml recombinant human IL-2 (Roche). Cells were washed twice with FACS buffer, stained with 1 µg/ml anti-CD8-PerCP Cy5.5 mAb for 30 mins on ice, fixed, permeabilised using the BD Cytofix/Cytoperm kit and stained with 5 µg/ml anti-IFN-γ-FITC, 2 µg/ml anti-TNF-α-APC, and 2 µg/ml anti-IL-2-PE mAbs. Samples were acquired by flow cytometry using a FACS Calibur and analysed by Flowjo. The total cytokine production was calculated by subtracting background fluorescence using no peptide controls.

### Isolation of single-cell tetramer-specific CD8^+^ T cells, RT-PCR and CDR3β sequencing

Splenocytes were stained with D^b^NP_366_-PE or D^b^PA_224_-PE tetramers in sort buffer (PBS with 0.1% BSA) for 1 hr at room temperature, washed and stained with 1 µg/ml anti-CD8-APC and 5 µg/ml of either anti-Vβ8.3 or anti-Vβ7-FITC for 30 mins on ice, washed twice with sort buffer. Single lymphocytes were isolated by sorting with a FACS Aria (BD Immunocytometry) into 80 wells of an empty 96 well twin-tec plate (Eppendorf). mRNA transcripts were reversed transcribed to cDNA, using a Sensiscript kit (Qiagen) according to manufacturer's instructions, and the CDR3β region amplified by a nested PCR using Vβprimers [44], [47], [57]. Positive PCR products were purified using QIAGEN PCR purification kit and sequenced.

### Statistical analysis

Magnitude, phenotype and function were compared between experimental aged and young groups by an unpaired Student's *t* test. Clonotypic diversity was quantified using both the number of different clonotypes and Simpson's diversity index. The overlap of TCRβ repertoires between mice was quantified using both the proportion of the TCRβ repertoires per mouse comprised of shared clonotypes and the Morisita-Horn similarity index. The Simpson's diversity and Morisita-Horn similarity indices account for both the variety of distinct clonotypes (defined either at the level of the amino acid or nucleotide sequence) and the clone size (number of copies) of each clonotype involved in the epitope-specific response within each mouse [48], [62]. The Simpson's diversity and Morisita-Horn similarity indices vary between 0 (minimum diversity/similarity) and 1 (maximum diversity/similarity). The diversity and similarity measures were calculated in conjunction with a randomization procedure to correct for differences in sample sizes between mice [48], [62], and were estimated for a subsample of 22 TCRβ sequences. To estimate the proportion of the TCRβ repertoires per mouse comprised of shared clonotypes, clonotypes were pre-defined as shared based on their presence in more than one mouse prior to the random subsampling of 22 sequences. A Mann-Whitney test was used to compare, between pairs of groups, the diversity (and similarity) between the aged and young groups of mice in primary responses and between young and aged (primed-young) and aged (primed-old) in secondary responses. Bonferroni correction for multiple pairwise comparisons was applied for the comparisons between the three secondary response groups (i.e. each pairwise test was assessed at the significance level of α = 0.05/3 = 0.0167). All statistical analyses were performed using GraphPad Prism version 5.04 (GraphPad Software Inc, San Diego, CA).

## Supporting Information

Figure S1 **Analysis of acute and memory CD8^+^ T cell responses elicited by i.p. priming.** Naïve B6 mice were i.p. primed with 1.5×10^7^ pfu of the PR8 virus. Influenza-specific CD8^+^ T cell responses were analysed in the spleen at the acute (d10), early memory (d23) and late memory (10 mths) phases of infection. (A) Total numbers of tetramer^+^CD8^+^ T cells are shown for immunodominant D^b^NP_366_^+^CD8^+^ and D^b^PA_224_^+^CD8^+^ T cell responses. (B) The contribution of immunodominant D^b^NP_366_^+^CD8^+^, D^b^PA_224_^+^CD8^+^ T cell responses in comparison with subdominant D^b^PB1_703_^+^CD8^+^ and K^b^PB1-F2_62_^+^CD8^+^ sets were calculated based on the proportions of IFN-γ^+^CD8^+^ populations. (C, D) Polyfunctionality of influenza-specific CD8^+^ T cell responses was assessed by simultaneous production of IFN-γ, TNF-α and IL-2.
(TIF)

Figure S2 **The expression of activation markers on influenza-specific CD8^+^ T cells in young and aged mice.** Phenotypic analysis of (A, D) CD27 vs CD43, (B, E) KLRG1 vs IL-7R, and (C, F) CD62L vs IL7-R was determined at the acute day 8 secondary time-point for (A–C) D^b^NP_366_, and (D–F) D^b^PA_224_ splenocytes from aged mice primed at 3 months and challenged at 22 months in comparison to young animals. Similar phenotypic data were obtained when aged mice were either primed at 22 months (primary response) or primed when young (at 6 weeks) and challenged at 22 months (secondary response). Data represent the mean ± SD of 3–5 mice per group. * = p<0.05.
(TIF)

Figure S3 **Comparison between aged and young mice of the characteristics of the D^b^NP_366_^+^CD8^+^ Vβ8.3^+^ and D^b^PA_224_^+^CD8^+^ Vβ7^+^ TCR repertoires during primary and secondary (primed-young and primed-old) infections.** The distributions of Jβ gene usage (A, B, E, F) and CDR3β length (C, D, G, H) among all D^b^NP_366_^+^CD8^+^ Vβ8.3^+^ TCR sequences during primary (A, C) and secondary (B, D) infections and all D^b^PA_224_^+^CD8^+^ Vβ7^+^ TCR sequences during primary (E, G) and secondary (F, H) infections.
(TIF)

Table S1 Nucleotide and amino acid CDR3β diversity profiles for primary D^b^NP_366_^+^Vβ8.3^+^CD8^+^ T cells in the aged (≥22months) mice.
(DOC)

Table S2 CDR3β diversity profiles for primary D^b^PA_224_^+^Vβ7^+^CD8^+^ T cells in the aged (≥22months) mice.
(DOC)

Table S3 Nucleotide and amino acid CDR3β diversity profiles for secondary D^b^NP_366_^+^Vβ8.3^+^CD8^+^ T cells in the aged (primed at 2months->challenged at 24 months) mice.
(DOC)

Table S4 CDR3β diversity profiles for secondary D^b^PA_224_^+^Vβ7^+^CD8^+^ T cells in the aged (primed at 2months,->challenged at 24 months) mice.
(DOC)

Table S5 Nucleotide and amino acid CDR3β diversity profiles for secondary D^b^NP_366_^+^Vβ8.3^+^CD8^+^ T cells in the aged (primed at ≥22months, challenged 6 weeks later) mice.
(DOC)

Table S6 Amino acid CDR3β diversity profiles for secondary D^b^PA_224_^+^Vβ7^+^CD8^+^ T cells in the aged (primed at ≥22months, challenged 6 weeks later) mice.
(DOC)
