# Correction: Early Priming Minimizes the Age-Related Immune Compromise of CD8^+^ T Cell Diversity and Function

**DOI:** 10.1371/annotation/e142f9de-7f30-4759-bda1-a651e86d5ba6

**Published:** 2012-03-15

**Authors:** Sophie A. Valkenburg, Vanessa Venturi, Thurston H. Y. Dang, Nicola L. Bird, Peter C. Doherty, Stephen J. Turner, Miles P. Davenport, Katherine Kedzierska

There was an error in affiliation #2 for authors Vanessa Venturi and Thurston H. Y. Dang. Affiliation 2 should be: Computational Biology Group, Centre for Vascular Research, University of New South Wales, Kensington, Australia.

