# Supplementary figures and images for: Early Priming Minimizes the Age-Related Immune Compromise of CD8+ T Cell Diversity and Function
Source: PLoS Pathog. 2012 Feb 23;8(2):e1002544. doi: 10.1371/journal.ppat.1002544 (PMC3285595; doi:10.1371/journal.ppat.1002544)

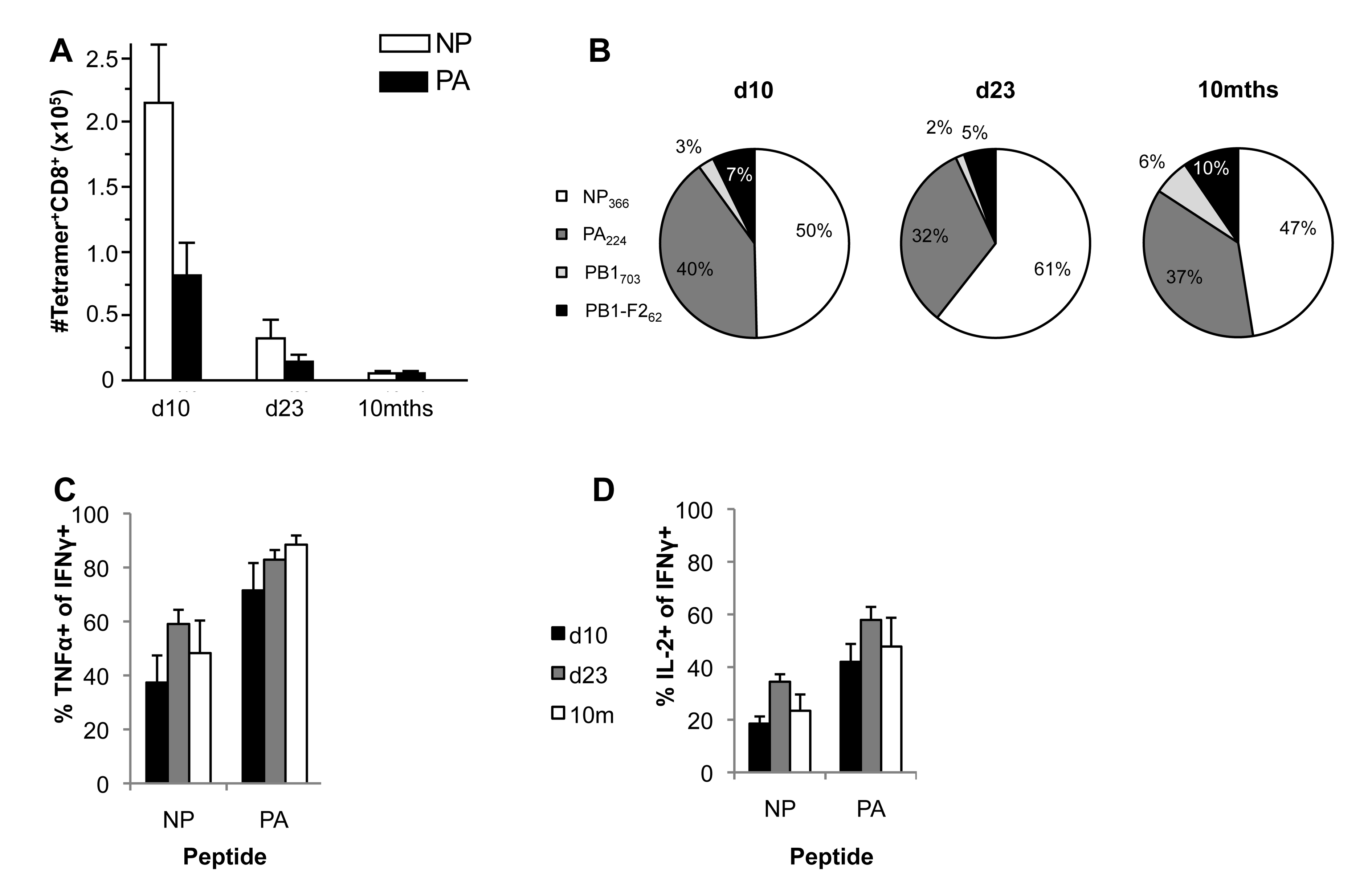

Supplement: Figure S1 — Analysis of acute and memory CD8+ T cell responses elicited by i.p. priming. Naïve B6 mice were i.p. primed with 1.5×10^7^ pfu of the PR8 virus. Influenza-specific CD8+ T cell responses were analysed in the spleen at the acute (d10), early memory (d23) and late memory (10 mths) phases of infection. (A) Total numbers of tetramer+CD8+ T cells are shown for immunodominant DbNP366 +CD8+ and DbPA224 +CD8+ T cell responses. (B) The contribution of immunodominant DbNP366 +CD8+, DbPA224 +CD8+ T cell responses in comparison with subdominant DbPB1703 +CD8+ and KbPB1-F262 +CD8+ sets were calculated based on the proportions of IFN-γ+CD8+ populations. (C, D) Polyfunctionality of influenza-specific CD8+ T cell responses was assessed by simultaneous production of IFN-γ, TNF-α and IL-2. (TIF) [file ppat.1002544.s001.tif]

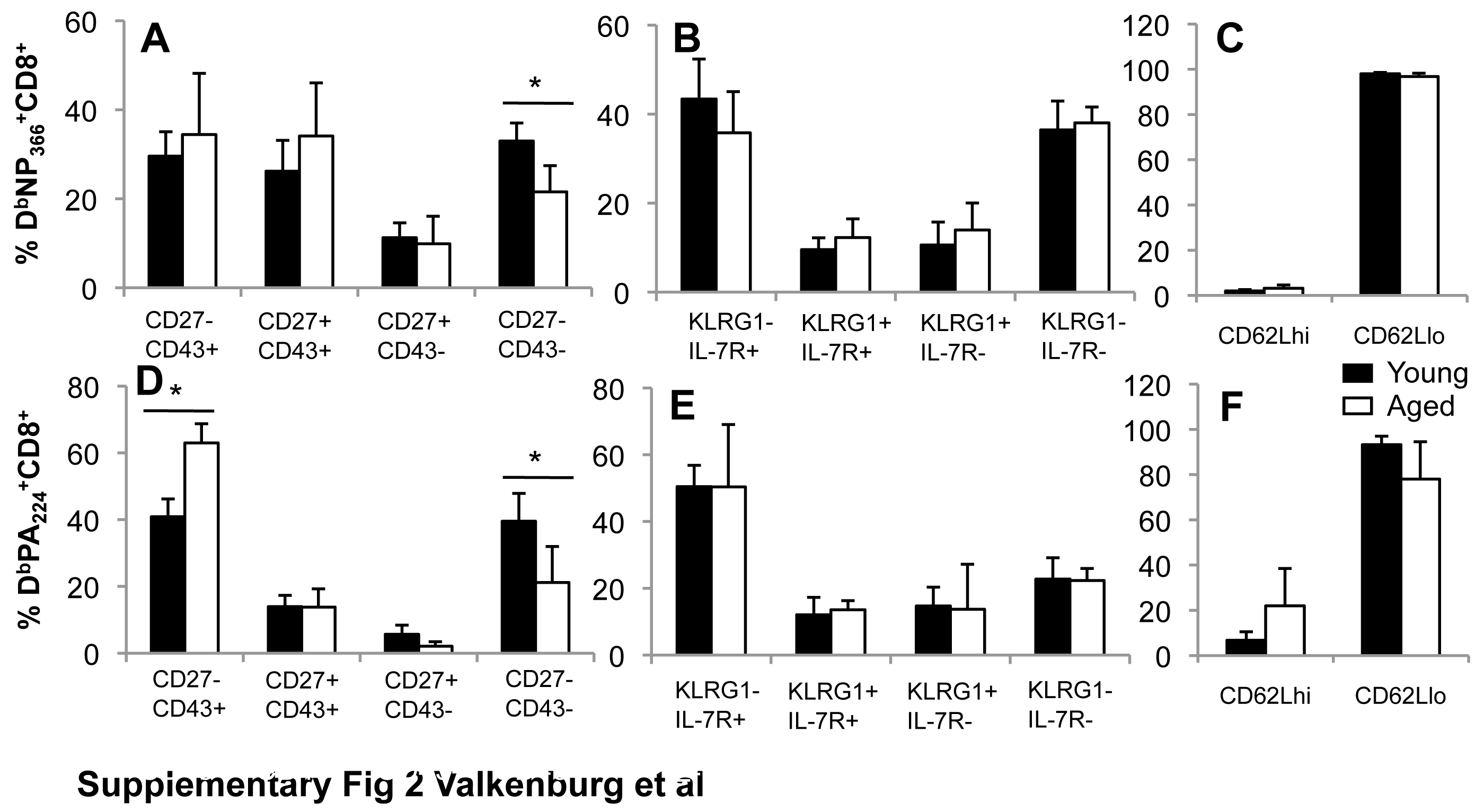

Supplement: Figure S2 — The expression of activation markers on influenza-specific CD8+ T cells in young and aged mice. Phenotypic analysis of (A, D) CD27 vs CD43, (B, E) KLRG1 vs IL-7R, and (C, F) CD62L vs IL7-R was determined at the acute day 8 secondary time-point for (A–C) DbNP366, and (D–F) DbPA224 splenocytes from aged mice primed at 3 months and challenged at 22 months in comparison to young animals. Similar phenotypic data were obtained when aged mice were either primed at 22 months (primary response) or primed when young (at 6 weeks) and challenged at 22 months (secondary response). Data represent the mean ± SD of 3–5 mice per group. * = p<0.05. (TIF) [file ppat.1002544.s002.tif]

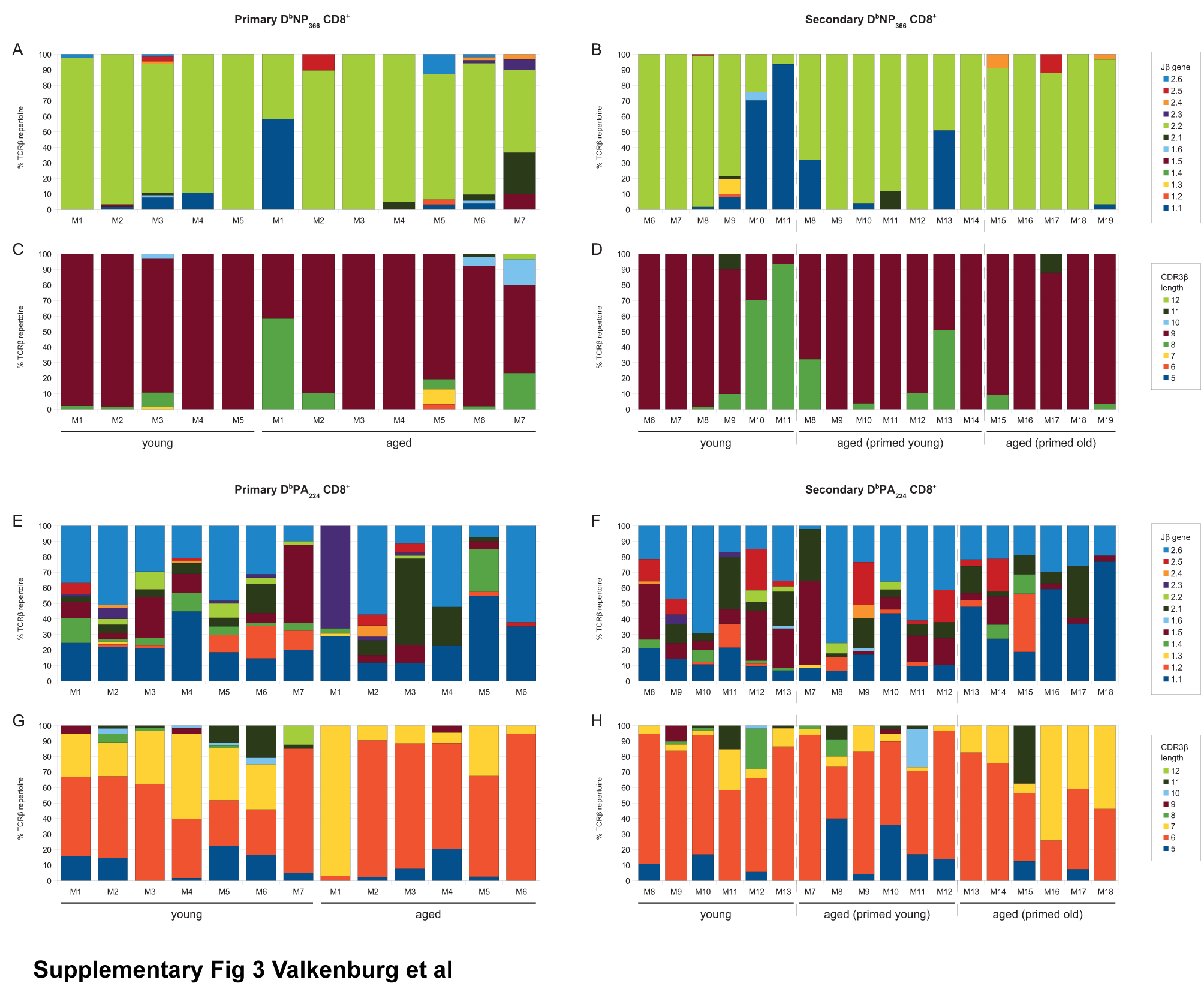

Supplement: Figure S3 — Comparison between aged and young mice of the characteristics of the DbNP366+CD8+ Vβ8.3+ and DbPA224+CD8+ Vβ7+ TCR repertoires during primary and secondary (primed-young and primed-old) infections. The distributions of Jβ gene usage (A, B, E, F) and CDR3β length (C, D, G, H) among all DbNP366 +CD8+ Vβ8.3+ TCR sequences during primary (A, C) and secondary (B, D) infections and all DbPA224 +CD8+ Vβ7+ TCR sequences during primary (E, G) and secondary (F, H) infections. (TIF) [file ppat.1002544.s003.tif]
