# Supplementary material for: Early Priming Minimizes the Age-Related Immune Compromise of CD8+ T Cell Diversity and Function
Source: PLoS Pathog. 2012 Feb 23;8(2):e1002544. doi: 10.1371/journal.ppat.1002544 (PMC3285595; doi:10.1371/journal.ppat.1002544)
Supplement: Table S1 — Nucleotide and amino acid CDR3β diversity profiles for primary DbNP366 +Vβ8.3+CD8+ T cells in the aged (≥22months) mice. (DOC) [file ppat.1002544.s004.doc]

Supplementary Table 1: Nucleotide and amino acid CDR3β diversity profiles for primary D^b^NP_366_^+^Vβ8.3^+^CD8^+^ T cells in the aged (≥22months) mice.

|  |  |  | **Frequency (%)** | | | | | | |
| --- | --- | --- | --- | --- | --- | --- | --- | --- | --- |
| **CDR3β seq** | **Jβ** | **aa length** | **M1** | **M2** | **M3** | **M4** | **M5** | **M6** | **M7** |
| **SGGANTGQL** | 2.2 | 9 |  |  |  |  |  |  |  |
| AGTGGCGGGGCAAACACCGGGCAGCTC |  |  |  | 90 |  | 95 |  |  |  |
| AGTGGGGGGGCCAACACCGGGCAGCTC |  |  |  |  |  |  | 77 |  | 40 |
| AGTGGGGGAGCAAACACCGGGCAGCTC |  |  |  |  | 53 |  |  |  |  |
| AGTGGGGGGGCAAACACCGGGCAGCTC |  |  | 39 |  |  |  |  |  |  |
| AGCGGGGGGGCAAACACCGGGCAGCTC |  |  | 3 |  |  |  |  |  |  |
| **SGGSNTGQL** | 2.2 | 9 |  |  |  |  |  |  |  |
| AGTGGAGGGTCAAACACCGGGCAGCTC |  |  |  |  |  |  |  | 33 |  |
| AGTGGCGGGTCCAACACCGGGCAGCTC |  |  |  |  |  |  | 3 |  |  |
| AGTGGGGGATCCAACACCGGGCAGCTC |  |  |  |  |  |  |  |  | 3 |
| AGTGGGGGGTCAAACACCGGGCAGCTC |  |  |  |  |  |  |  |  | 3 |
| AGTGGGGGGTCCAACACCGGGCAGCTC |  |  |  |  |  |  |  |  | 3 |
| **SGRKNTEV** | 1.1 | 8 |  |  |  |  |  |  |  |
| TCGGGACGTAAAAACACAGAAGTC |  |  | 58 |  |  |  |  |  |  |
| **KGGGNTGQL** | 2.2 | 9 |  |  |  |  |  |  |  |
| AAAGGGGGGGGGAACACCGGGCAGCTC |  |  |  |  |  |  |  | 42 |  |
| **RGGGNTGQL** | 2.2 | 9 |  |  |  |  |  |  |  |
| AGGGGGGGGGGAAACACCGGGCAGCTC |  |  |  |  | 27 |  |  |  |  |
| **RGGPYAEQ** | 2.1 | 8 |  |  |  |  |  |  |  |
| AGGGGGGGGCCCTATGCTGAGCAG |  |  |  |  |  |  |  |  | 23 |
| **RGGSNTGQL** | 2.2 | 9 |  |  |  |  |  |  |  |
| AGGGGGGGGTCAAACACCGGGCAGCTC |  |  |  |  | 11 |  |  |  |  |
| **STGGRDTQ** | 2.5 | 8 |  |  |  |  |  |  |  |
| AGCACTGGGGGGCGAGACACCCAG |  |  |  | 10 |  |  |  |  |  |
| **RGGANTGQL** | 2.2 | 9 |  |  |  |  |  |  |  |
| AGGGGTGGGGCAAACACCGGGCAGCTC |  |  |  |  |  |  |  | 10 |  |
| **SRDKYNNQAP** | 1.5 | 10 |  |  |  |  |  |  |  |
| AGTCGGGACAAATATAACAACCAGGCTCCG |  |  |  |  |  |  |  |  | 10 |
| **KGGSNTGQL** | 2.2 | 9 |  |  |  |  |  |  |  |
| AAGGGGGGCTCAAACACCGGGCAGCTC |  |  |  |  | 9 |  |  |  |  |
| **SDGWGAQ** | 2.6 | 7 |  |  |  |  |  |  |  |
| AGTGACGGCTGGGGGGCACAG |  |  |  |  |  |  | 6 |  |  |
| **SGGANYAEQ** | 2.1 | 9 |  |  |  |  |  |  |  |
| AGTGGAGGGGCTAACTATGCTGAGCAG |  |  |  |  |  | 5 |  |  |  |
| **SDHRGRTEV** | 1.1 | 9 |  |  |  |  |  |  |  |
| AGTGACCACAGGGGGCGAACAGAAGTC |  |  |  |  |  |  |  | 4 |  |
| **RLLSSAETL** | 2.3 | 9 |  |  |  |  |  |  |  |
| AGGCTCCTTTCTAGTGCAGAAACGCTG |  |  |  |  |  |  |  |  | 3 |
| **SATGGGDAEQ** | 2.1 | 10 |  |  |  |  |  |  |  |
| AGTGCGACTGGGGGGGGCGATGCTGAGCAG |  |  |  |  |  |  |  |  | 3 |
| **SDEDWGVSQNTL** | 2.4 | 12 |  |  |  |  |  |  |  |
| AGTGATGAGGACTGGGGGGTTAGTCAAAACACCTTG |  |  |  |  |  |  |  |  | 3 |
| **SDGGEQ** | 2.6 | 6 |  |  |  |  |  |  |  |
| AGTGATGGGGGGGAACAG |  |  |  |  |  |  | 3 |  |  |
| **SDKNSDY** | 1.2 | 7 |  |  |  |  |  |  |  |
| AGTGACAAAAACTCCGACTAC |  |  |  |  |  |  | 3 |  |  |
| **SDRGNTEV** | 1.1 | 8 |  |  |  |  |  |  |  |
| AGTGACAGGGGGAACACAGAAGTC |  |  |  |  |  |  | 3 |  |  |
| **SDSGGAGQ** | 2.6 | 8 |  |  |  |  |  |  |  |
| AGTGATTCTGGGGGGGCGGGACAG |  |  |  |  |  |  | 3 |  |  |
| **SDTGVSAETL** | 2.3 | 10 |  |  |  |  |  |  |  |
| AGCGATACTGGGGTCAGTGCAGAAACGCTG |  |  |  |  |  |  |  |  | 3 |
| **SGGPNTGQL** | 2.2 | 9 |  |  |  |  |  |  |  |
| AGTGGGGGGCCAAACACCGGGCAGCTC |  |  |  |  |  |  |  |  | 3 |
| **SGGTGGYAEQ** | 2.1 | 10 |  |  |  |  |  |  |  |
| AGTGGCGGGACAGGGGGATATGCTGAGCAG |  |  |  |  |  |  |  | 2 |  |
| **SPGLGGPYEQ** | 2.6 | 10 |  |  |  |  |  |  |  |
| AGTCCGGGACTGGGGGGTCCCTATGAACAG |  |  |  |  |  |  |  | 2 |  |
| **SPPDSAETL** | 2.3 | 9 |  |  |  |  |  |  |  |
| AGTCCTCCAGACAGTGCAGAAACGCTG |  |  |  |  |  |  |  | 2 |  |
| **SDRDRYNSPL** | 1.6 | 10 |  |  |  |  |  |  |  |
| AGTGACAGGGACAGGTATAATTCGCCCCTC |  |  |  |  |  |  |  | 2 |  |
| **SDGDWGGQNTL** | 2.4 | 11 |  |  |  |  |  |  |  |
| AGTGATGGGGACTGGGGCGGTCAAAACACCTTG |  |  |  |  |  |  |  | 2 |  |
| **SVGGRAEQ** | 2.1 | 8 |  |  |  |  |  |  |  |
| AGTGTGGGGGGGCGGGCTGAGCAG |  |  |  |  |  |  |  | 2 |  |
|  |  |  | **36** | **48** | **45** | **42** | **31** | **52** | **30** |

M: individual mouse
