# Supplementary material for: Early Priming Minimizes the Age-Related Immune Compromise of CD8+ T Cell Diversity and Function
Source: PLoS Pathog. 2012 Feb 23;8(2):e1002544. doi: 10.1371/journal.ppat.1002544 (PMC3285595; doi:10.1371/journal.ppat.1002544)
Supplement: Table S2 — CDR3β diversity profiles for primary DbPA224 +Vβ7+CD8+ T cells in the aged (≥22months) mice. (DOC) [file ppat.1002544.s005.doc]

Supplementary Table 2: CDR3β diversity profiles for primary D^b^PA_224_^+^Vβ7^+^CD8^+^ T cells in the aged (≥22months) mice.

|  |  |  | **Frequency (%)** | | | | | |
| --- | --- | --- | --- | --- | --- | --- | --- | --- |
| **CDR3β** | **Jβ** | **aa length** | **M1** | **M2** | **M3** | **M4** | **M5** | **M6** |
| SLGGEV | 1.1 | 6 |  | 12 |  | 2 |  | 30 |
| SLGSEQ | 2.6 | 6 |  |  |  | 11 |  | 62 |
| SSYEQ | 2.6 | 5 |  | 2 | 8 |  |  |  |
| SLGEDTQ | 2.5 | 7 |  |  | 6 |  |  | 3 |
| SSGQGAG | 2.3 | 7 | 66 |  |  |  |  |  |
| SPGDEQ | 2.6 | 6 |  | 52 |  |  |  |  |
| SYGSEQ | 2.1 | 6 |  |  | 52 |  |  |  |
| SADRGEV | 1.1 | 7 | 29 |  |  |  |  |  |
| SPDRGQV | 1.1 | 7 |  |  |  |  | 23 |  |
| SSGERL | 1.4 | 6 |  |  |  |  | 23 |  |
| SEAEQ | 2.1 | 5 |  |  |  | 20 |  |  |
| SWGDEQ | 2.6 | 6 |  |  |  | 20 |  |  |
| SSGDEQ | 2.6 | 6 |  |  |  | 18 |  |  |
| SEGPEV | 1.1 | 6 |  |  |  |  | 13 |  |
| SEGSEQ | 2.1 | 6 |  | 10 |  |  |  |  |
| SSGKEV | 1.1 | 6 |  |  |  |  | 10 |  |
| SSGTEV | 1.1 | 6 |  |  |  | 9 |  |  |
| SFGGEV | 1.1 | 6 |  |  | 8 |  |  |  |
| SEDRGTL | 2.4 | 7 |  | 7 |  |  |  |  |
| TQGDTQ | 2.5 | 6 |  | 7 |  |  |  |  |
| SFSTEV | 1.1 | 6 |  |  |  | 7 |  |  |
| SWGEAP | 1.5 | 6 |  |  | 6 |  |  |  |
| SFGQAP | 1.5 | 6 |  | 5 |  |  |  |  |
| SLDRGQV | 1.1 | 7 |  |  |  |  | 5 |  |
| SQGGEV | 1.1 | 6 |  |  |  |  | 5 |  |
| TGGGAP | 1.5 | 6 |  |  | 4 |  |  |  |
| SLGERL | 1.4 | 6 | 3 |  |  |  |  |  |
| RGSDY | 1.2 | 5 |  |  |  |  | 3 |  |
| SFGAPQ | 2.1 | 6 |  |  |  |  | 3 |  |
| SFGEGQ | 2.6 | 6 |  |  |  |  | 3 |  |
| SGDGQAP | 1.5 | 7 |  |  |  |  | 3 |  |
| SGGDEQ | 2.6 | 6 |  |  |  |  | 3 |  |
| SLSPERL | 1.4 | 7 |  |  |  |  | 3 |  |
| SSGKAP | 1.5 | 6 |  |  |  |  | 3 |  |
| SWGERL | 1.4 | 6 |  |  |  |  | 3 |  |
| TGGGEQ | 2.6 | 6 |  |  |  |  | 3 |  |
| SPDRGHV | 1.1 | 7 |  |  |  |  |  | 3 |
| SSGREV | 1.1 | 6 |  |  |  |  |  | 3 |
| SWDRGTL | 1.3 | 7 | 2 |  |  |  |  |  |
| DWGDEQ | 2.6 | 6 |  | 2 |  |  |  |  |
| TGTPETL | 2.3 | 7 |  | 2 |  |  |  |  |
| SAPTEV | 1.1 | 6 |  |  | 2 |  |  |  |
| SLDRGQL | 2.2 | 7 |  |  | 2 |  |  |  |
| SLGAEQ | 2.1 | 6 |  |  | 2 |  |  |  |
| SLGGAP | 1.5 | 6 |  |  | 2 |  |  |  |
| SQGGEQ | 2.6 | 6 |  |  | 2 |  |  |  |
| SSAETL | 2.3 | 6 |  |  | 2 |  |  |  |
| SWDRGEV | 1.1 | 7 |  |  | 2 |  |  |  |
| SWGGEEQ | 2.6 | 7 |  |  | 2 |  |  |  |
| TWGAEQ | 2.1 | 6 |  |  | 2 |  |  |  |
| SFRNTEV | 1.1 | 7 |  |  |  | 2 |  |  |
| SFSGGRAEQ | 2.1 | 9 |  |  |  | 2 |  |  |
| SLKGVEQ | 2.6 | 7 |  |  |  | 2 |  |  |
| SLRTGWAEQ | 2.1 | 9 |  |  |  | 2 |  |  |
| STGSTEV | 1.1 | 7 |  |  |  | 2 |  |  |
| **Total sequences** |  |  | **62** | **42** | **52** | **44** | **40** | **37** |
