# Supplementary material for: Early Priming Minimizes the Age-Related Immune Compromise of CD8+ T Cell Diversity and Function
Source: PLoS Pathog. 2012 Feb 23;8(2):e1002544. doi: 10.1371/journal.ppat.1002544 (PMC3285595; doi:10.1371/journal.ppat.1002544)
Supplement: Table S3 — Nucleotide and amino acid CDR3β diversity profiles for secondary DbNP366 +Vβ8.3+CD8+ T cells in the aged (primed at 2months->challenged at 24 months) mice. (DOC) [file ppat.1002544.s006.doc]

Supplementary Table 3: Nucleotide and amino acid CDR3β diversity profiles for secondary D^b^NP_366_^+^Vβ8.3^+^CD8^+^ T cells in the aged (primed at 2months->challenged at 24 months) mice.

|  |  |  | **Frequency (%)** | | | | | | |
| --- | --- | --- | --- | --- | --- | --- | --- | --- | --- |
| **CDR3β seq** | **Jβ** | **aa length** | **M8** | **M9** | **M10** | **M11** | **M12** | **M13** | **M14** |
| **SGGANTGQL** | 2.2 | 9 |  |  |  |  |  |  |  |
| AGTGGGGGGGCAAACACCGGGCAGCTC |  |  |  | 2 | 6 | 4 |  |  |  |
| AGTGGGGGAGCAAACACCGGGCAGCTC |  |  |  |  |  |  |  |  | 36 |
| AGTGGGGGCGCAAACACCGGGCAGCTC |  |  |  | 33 |  |  |  |  |  |
| TCTGGGGGAGCAAACACCGGGCAGCTC |  |  |  |  |  |  |  |  | 25 |
| TCCGGGGGGGCAAACACCGGGCAGCTC |  |  |  |  |  |  |  |  | 22 |
| AGTGGGGGGGCCAACACCGGGCAGCTC |  |  |  |  |  |  |  |  | 17 |
| AGTGGGGGGGCGAACACCGGGCAGCTC |  |  |  |  | 6 |  |  |  |  |
| AGTGGGGGTGCAAACACCGGGCAGCTC |  |  | 4 |  |  |  |  |  |  |
| AGCGGGGGGGCAAACACCGGGCAGCTC |  |  |  |  | 2 |  |  |  |  |
| **SGGSNTGQL** | 2.2 | 9 |  |  |  |  |  |  |  |
| AGTGGGGGGTCAAACACCGGGCAGCTC |  |  |  |  |  | 68 | 67 |  |  |
| AGTGGGGGATCAAACACCGGGCAGCTC |  |  | 43 |  |  |  | 2 |  |  |
| AGTGGTGGGTCAAACACCGGGCAGCTC |  |  |  |  |  | 16 |  |  |  |
| AGTGGGGGCTCAAACACCGGGCAGCTC |  |  |  |  |  |  |  | 2 |  |
| **SGGGNTGQL** | 2.2 | 9 |  |  |  |  |  |  |  |
| AGTGGGGGGGGGAATACCGGGCAGCTC |  |  |  | 65 |  |  |  |  |  |
| AGTGGGGGTGGCAACACCGGGCAGCTC |  |  | 18 |  |  |  |  |  |  |
| AGTGGGGGGGGGAACACCGGGCAGCTC |  |  |  |  | 2 |  |  |  |  |
| **SGGGRTGQL** | 2.2 | 9 |  |  |  |  |  |  |  |
| AGTGGGGGGGGGCGCACCGGGCAGCTC |  |  |  |  | 79 |  |  |  |  |
| AGTGGCGGGGGGCGCACCGGGCAGCTC |  |  | 4 |  |  |  |  |  |  |
| **SDAAATEV** | 1.1 | 8 |  |  |  |  |  |  |  |
| AGTGATGCGGCAGCCACAGAAGTC |  |  |  |  |  |  |  | 51 |  |
| **KGGGNTGQL** | 2.2 | 9 |  |  |  |  |  |  |  |
| AAGGGAGGGGGAAACACCGGGCAGCTC |  |  |  |  |  |  |  | 40 |  |
| **RDSANTEV** | 1.1 | 8 |  |  |  |  |  |  |  |
| AGGGACAGTGCAAACACAGAAGTC |  |  | 25 |  |  |  |  |  |  |
| **KGGARTGQL** | 2.2 | 9 |  |  |  |  |  |  |  |
| AAGGGGGGGGCGCGGACCGGGCAGCTC |  |  |  |  |  |  | 21 |  |  |
| **RAGGNYAEQ** | 2.1 | 9 |  |  |  |  |  |  |  |
| AGGGCTGGGGGTAACTATGCTGAGCAG |  |  |  |  |  | 12 |  |  |  |
| **SDWYTGQL** | 2.2 | 8 |  |  |  |  |  |  |  |
| AGTGACTGGTACACCGGGCAGCTC |  |  |  |  |  |  | 10 |  |  |
| **SDAANTEV** | 1.1 | 8 |  |  |  |  |  |  |  |
| AGTGATGCGGCCAACACAGAAGTC |  |  | 7 |  |  |  |  |  |  |
| **RGGANTGQL** | 2.2 | 9 |  |  |  |  |  |  |  |
| AGAGGGGGGGCAAACACCGGGCAGCTC |  |  |  |  |  |  |  | 5 |  |
| **SDLGKTEV** | 1.1 | 8 |  |  |  |  |  |  |  |
| AGTGATCTGGGAAAAACAGAAGTC |  |  |  |  | 4 |  |  |  |  |
| **SGGGGTRQL** | 2.2 | 9 |  |  |  |  |  |  |  |
| AGTGGGGGGGGGGGCACCCGGCAACTC |  |  |  |  | 2 |  |  |  |  |
| **NGGANTGQL** | 2.2 | 9 |  |  |  |  |  |  |  |
| AATGGGGGGGCAAACACCGGGCAGCTC |  |  |  |  |  |  |  | 2 |  |
|  |  |  | **28** | **55** | **53** | **50** | **58** | **55** | **59** |

M: Individual mouse
