# Supplementary material for: Early Priming Minimizes the Age-Related Immune Compromise of CD8+ T Cell Diversity and Function
Source: PLoS Pathog. 2012 Feb 23;8(2):e1002544. doi: 10.1371/journal.ppat.1002544 (PMC3285595; doi:10.1371/journal.ppat.1002544)
Supplement: Table S4 — CDR3β diversity profiles for secondary DbPA224 +Vβ7+CD8+ T cells in the aged (primed at 2months,->challenged at 24 months) mice. (DOC) [file ppat.1002544.s007.doc]

Supplementary Table 4. CDR3β diversity profiles for secondary D^b^PA_224_^+^Vβ7^+^CD8^+^ T cells in the aged (primed at 2months,->challenged at 24 months) mice.

|  |  |  | **Frequency (%)** | | | | | |
| --- | --- | --- | --- | --- | --- | --- | --- | --- |
| **CDR3β** | **Jβ** | **aa length** | **M7** | **M8** | **M9** | **M10** | **M11** | **M12** |
| SSYEQ | 2.6 | 5 |  | 24 | 2 | 33 | 12 | 10 |
| SLGAEQ | 2.1 | 6 |  |  | 19 |  | 2 | 3 |
| SAAEQ | 2.1 | 5 |  |  |  | 3 | 5 | 3 |
| SFGGEV | 1.1 | 6 |  |  |  | 13 | 2 |  |
| SYGGEQ | 2.6 | 6 |  |  |  |  | 2 | 10 |
| GSYEQ | 2.6 | 5 |  | 9 | 2 |  |  |  |
| SWGSEQ | 2.6 | 6 |  |  | 4 |  |  | 3 |
| SLGGGV | 1.1 | 6 | 4 |  | 2 |  |  |  |
| SLTGEQ | 2.6 | 6 |  |  | 4 |  | 2 |  |
| SFGKAP | 1.5 | 6 | 48 |  |  |  |  |  |
| TGGAEQ | 2.1 | 6 | 33 |  |  |  |  |  |
| SRGGEV | 1.1 | 6 |  |  |  | 28 |  |  |
| TSGDTQ | 2.5 | 6 |  |  | 26 |  |  |  |
| SFGGEQ | 2.6 | 6 |  | 24 |  |  |  |  |
| SPPDWESYEQ | 2.6 | 10 |  |  |  |  | 24 |  |
| SLGDTQ | 2.5 | 6 |  |  |  |  |  | 21 |
| SFGQAP | 1.5 | 6 |  |  |  |  | 17 |  |
| TTGGEQ | 2.6 | 6 |  |  |  |  | 15 |  |
| SRGGAP | 1.5 | 6 |  |  |  |  |  | 14 |
| SLGGRV | 1.1 | 6 |  |  | 11 |  |  |  |
| CGGGEQ | 2.6 | 6 |  |  |  |  |  | 10 |
| SLSHRGRNSDY | 1.2 | 11 |  | 9 |  |  |  |  |
| SFDRGAL | 2.4 | 7 |  |  | 9 |  |  |  |
| IGSNTGQL | 2.2 | 8 |  | 7 |  |  |  |  |
| SAGAEV | 1.1 | 6 |  |  |  |  | 7 |  |
| SGGGEQ | 2.6 | 6 |  |  |  |  |  | 7 |
| SQGAEV | 1.1 | 6 |  |  |  |  |  | 7 |
| AGGQAP | 1.5 | 6 | 6 |  |  |  |  |  |
| SFDRGQL | 2.2 | 7 |  |  |  | 5 |  |  |
| SGGQAP | 1.5 | 6 |  |  |  | 5 |  |  |
| RSYEQ | 2.6 | 5 |  | 4 |  |  |  |  |
| SLDRGEV | 1.1 | 7 |  | 4 |  |  |  |  |
| SLGGEV | 1.1 | 6 |  |  |  | 3 |  |  |
| MGAGDYAEQ | 2.1 | 9 |  |  |  | 3 |  |  |
| SEGDAP | 1.5 | 6 |  |  |  | 3 |  |  |
| SKGGEQ | 2.6 | 6 |  |  |  | 3 |  |  |
| SLSHRDRNSDY | 1.2 | 11 |  |  |  | 3 |  |  |
| CGGGEQ | 2.1 | 6 |  |  |  |  |  | 3 |
| SLGGPP | 1.5 | 6 |  |  |  |  |  | 3 |
| SPDRGEV | 1.1 | 7 |  |  |  |  |  | 3 |
| GGDTEV | 1.1 | 6 | 2 |  |  |  |  |  |
| RQGAGNTL | 1.3 | 8 | 2 |  |  |  |  |  |
| SLDRGHV | 1.1 | 7 | 2 |  |  |  |  |  |
| SPDRGEQ | 2.6 | 7 | 2 |  |  |  |  |  |
| GFGGEQ | 2.6 | 6 |  | 2 |  |  |  |  |
| IGTGGYEQ | 2.6 | 8 |  | 2 |  |  |  |  |
| RFGGEQ | 2.6 | 6 |  | 2 |  |  |  |  |
| RGTGGYEQ | 2.6 | 8 |  | 2 |  |  |  |  |
| SGGAEQ | 2.1 | 6 |  | 2 |  |  |  |  |
| SLDRGEQ | 2.6 | 7 |  | 2 |  |  |  |  |
| SQAEV | 1.1 | 5 |  | 2 |  |  |  |  |
| SWGDEQ | 2.6 | 6 |  | 2 |  |  |  |  |
| SDSTEV | 1.1 | 6 |  |  | 2 |  |  |  |
| SFDRGEQ | 2.6 | 7 |  |  | 2 |  |  |  |
| SLAGYEQ | 2.6 | 7 |  |  | 2 |  |  |  |
| SLGGEQ | 2.6 | 6 |  |  | 2 |  |  |  |
| SSPHEQ | 2.6 | 6 |  |  | 2 |  |  |  |
| SSPNEQ | 2.6 | 6 |  |  | 2 |  |  |  |
| SYGDSPL | 1.6 | 7 |  |  | 2 |  |  |  |
| SYGEAP | 1.5 | 6 |  |  | 2 |  |  |  |
| TGTGTEV | 1.1 | 7 |  |  | 2 |  |  |  |
| TSGDIQ | 2.5 | 6 |  |  | 2 |  |  |  |
| SLSWRGEDSDY | 1.2 | 11 |  |  |  |  | 2 |  |
| SSPDTQ | 2.5 | 6 |  |  |  |  | 2 |  |
| SWGYEQ | 2.6 | 6 |  |  |  |  | 2 |  |
| SYGSDEQ | 2.6 | 7 |  |  |  |  | 2 |  |
| **Total sequences** |  |  | **48** | **45** | **47** | **39** | **41** | **29** |
