# Supplementary material for: Early Priming Minimizes the Age-Related Immune Compromise of CD8+ T Cell Diversity and Function
Source: PLoS Pathog. 2012 Feb 23;8(2):e1002544. doi: 10.1371/journal.ppat.1002544 (PMC3285595; doi:10.1371/journal.ppat.1002544)
Supplement: Table S5 — Nucleotide and amino acid CDR3β diversity profiles for secondary DbNP366 +Vβ8.3+CD8+ T cells in the aged (primed at ≥22months, challenged 6 weeks later) mice. (DOC) [file ppat.1002544.s008.doc]

Supplementary Table 5: Nucleotide and amino acid CDR3β diversity profiles for secondary D^b^NP_366_^+^Vβ8.3^+^CD8^+^ T cells in the aged (primed at ≥22months, challenged 6 weeks later) mice.

|  |  |  | **Frequency (%)** | | | | |
| --- | --- | --- | --- | --- | --- | --- | --- |
| **CDR3β seq** | **Jβ** | **aa length** | **M1** | **M2** | **M3** | **M4** | **M5** |
| **SGGSNTGQL** | 2.2 | 9 |  |  |  |  |  |
| AGTGGGGGGTCAAACACCGGGCAGCTC |  |  | 82 | 3 | 52 | 9 | 13 |
| AGTGGCGGGTCTAACACCGGGCAGCTC |  |  |  |  |  | 82 | 73 |
| AGTGGGGGTTCAAACACCGGGCAGCTC |  |  |  |  | 15 |  |  |
| AGTGGAGGGTCAAACACCGGGCAGCTC |  |  |  |  | 15 |  |  |
| AGCGGGGGGTCAAACACCGGGCAGCTC |  |  |  |  |  | 9 |  |
| AGTGGGGGCTCAAACACCGGGCAGCTC |  |  | 3 |  |  |  |  |
| **SGGGNTGQL** | 2.2 | 9 |  |  |  |  |  |
| AGTGGGGGGGGAAACACCGGGCAGCTC |  |  | 6 | 3 |  |  |  |
| **RGGANTGQL** | 2.2 | 9 |  |  |  |  |  |
| AGAGGGGGGGCAAACACCGGGCAGCTC |  |  |  |  |  |  | 3 |
| AGGGGAGGAGCAAACACCGGGCAGCTC |  |  |  | 80 |  |  |  |
| **SGGANTGQL** | 2.2 | 9 |  |  |  |  |  |
| AGTGGGGGGGCCAACACCGGGCAGCTC |  |  |  |  | 6 |  |  |
| AGTGGGGGGGCGAACACCGGGCAGCTC |  |  |  |  |  |  | 3 |
| **KGGGNTGQL** | 2.2 | 9 |  |  |  |  |  |
| AAAGGGGGGGGAAACACCGGGCAGCTC |  |  |  | 14 |  |  |  |
| **SDAKGKRKDT** | 2.5 | 11 |  |  |  |  |  |
| AGTGATGCAAAGGGGAAGAGGAAAGACACCCAG |  |  |  |  | 12 |  |  |
| **SVGGRDT** | 2.4 | 8 |  |  |  |  |  |
| AGTGTAGGGGGGCGGGACACCTTG |  |  | 9 |  |  |  |  |
| **SDAQKTEV** | 1.1 | 8 |  |  |  |  |  |
| AGTGATGCACAAAAGACAGAAGTC |  |  |  |  |  |  | 3 |
| **SDWGWQNTL** | 2.4 | 9 |  |  |  |  |  |
| AGTGATTGGGGGTGGCAAAACACCTTG |  |  |  |  |  |  | 3 |
| **Total** |  |  | **33** | **35** | **33** | **22** | **30** |

M: individual mouse
