# Supplementary material for: Early Priming Minimizes the Age-Related Immune Compromise of CD8+ T Cell Diversity and Function
Source: PLoS Pathog. 2012 Feb 23;8(2):e1002544. doi: 10.1371/journal.ppat.1002544 (PMC3285595; doi:10.1371/journal.ppat.1002544)
Supplement: Table S6 — Amino acid CDR3β diversity profiles for secondary DbPA224 +Vβ7+CD8+ T cells in the aged (primed at ≥22months, challenged 6 weeks later) mice. (DOC) [file ppat.1002544.s009.doc]

Supplementary Table 6: Amino acid CDR3β diversity profiles for secondary DbPA_224_^+^Vβ7^+^CD8^+^ T cells in the aged (primed at ≥22months, challenged 6 weeks later) mice.

|  |  |  | **Frequency (%)** | | | | | |
| --- | --- | --- | --- | --- | --- | --- | --- | --- |
| **CDR3β** | **Jβ** | **aa length** | **M1** | **M2** | **M3** | **M4** | **M5** | **M6** |
| SLGNTEV | 1.1 | 7 | 13 |  |  | 56 | 33 | 35 |
| SYGNEQ | 2.6 | 6 |  |  |  | 7 | 4 | 12 |
| SFGAEQ | 2.6 | 6 | 4 | 3 |  |  | 4 |  |
| SLSGYEQ | 2.6 | 7 |  |  |  | 11 |  | 4 |
| SLDRGEV | 1.1 | 7 |  | 3 |  |  |  | 8 |
| SFGREQ | 2.6 | 6 |  |  |  | 4 | 7 |  |
| SAGREQ | 2.6 | 6 | 4 |  |  |  |  | 4 |
| TGGAEQ | 2.1 | 6 |  | 3 | 3 |  |  |  |
| SFGAEQ | 2.1 | 6 | 17 |  |  |  |  |  |
| QQGEGV | 1.1 | 6 | 9 |  |  |  |  |  |
| SSPGGQ | 2.6 | 6 | 9 |  |  |  |  |  |
| SLGGEV | 1.1 | 6 | 9 |  |  |  |  |  |
| RLGDTQ | 2.5 | 6 | 4 |  |  |  |  |  |
| RQGEEV | 1.1 | 6 | 4 |  |  |  |  |  |
| SFGEAP | 1.5 | 6 | 4 |  |  |  |  |  |
| SPDRGQV | 1.1 | 7 | 4 |  |  |  |  |  |
| SQGEEV | 1.1 | 6 | 4 |  |  |  |  |  |
| SRGGEV | 1.1 | 6 | 4 |  |  |  |  |  |
| TGGSDY | 1.2 | 6 | 4 |  |  |  |  |  |
| SSGGGQ | 2.6 | 6 | 4 |  |  |  |  |  |
| SWGEDTQ | 2.5 | 7 |  | 21 |  |  |  |  |
| SSGEAP | 1.5 | 6 |  | 18 |  |  |  |  |
| SSGDEQ | 2.6 | 6 |  | 12 |  |  |  |  |
| SWGAEV | 1.1 | 6 |  | 12 |  |  |  |  |
| SFGGGV | 1.1 | 6 |  | 6 |  |  |  |  |
| SSPERL | 1.4 | 6 |  | 6 |  |  |  |  |
| SQGAEV | 1.1 | 6 |  | 6 |  |  |  |  |
| SWTGEQ | 2.6 | 6 |  | 3 |  |  |  |  |
| SLGDEQ | 2.6 | 6 |  | 3 |  |  |  |  |
| SLGGRL | 1.4 | 6 |  | 3 |  |  |  |  |
| SSPYRGRDSDY | 1.2 | 11 |  |  | 38 |  |  |  |
| SGGGEQ | 2.6 | 6 |  |  | 16 |  |  |  |
| SGGEV | 1.1 | 5 |  |  | 13 |  |  |  |
| SLGDRE | 1.4 | 6 |  |  | 13 |  |  |  |
| SLGAEQ | 2.1 | 6 |  |  | 9 |  |  |  |
| RPDRGRG | 1.1 | 7 |  |  | 3 |  |  |  |
| SFGDEQ | 2.6 | 6 |  |  | 3 |  |  |  |
| SPDRGRV | 1.1 | 7 |  |  | 3 |  |  |  |
| QGGAEQ | 2.1 | 6 |  |  |  | 7 |  |  |
| SAGEAP | 1.5 | 6 |  |  |  | 4 |  |  |
| SLDGGEQ | 2.6 | 7 |  |  |  | 4 |  |  |
| SWDRGEV | 1.1 | 7 |  |  |  | 4 |  |  |
| SWGREQ | 2.6 | 6 |  |  |  | 4 |  |  |
| SPGAEQ | 2.1 | 6 |  |  |  |  | 30 |  |
| SSYEQ | 2.6 | 5 |  |  |  |  | 7 |  |
| DGFYAEQ | 2.1 | 7 |  |  |  |  | 4 |  |
| SLGGEQ | 2.6 | 6 |  |  |  |  | 4 |  |
| SLWNTEV | 1.1 | 7 |  |  |  |  | 4 |  |
| SSGQAP | 1.5 | 6 |  |  |  |  | 4 |  |
| SLGNEV | 1.1 | 6 |  |  |  |  |  | 15 |
| SGGTEV | 1.1 | 6 |  |  |  |  |  | 12 |
| SFGQAP | 1.5 | 6 |  |  |  |  |  | 4 |
| SPDRGKV | 1.1 | 7 |  |  |  |  |  | 4 |
| SWDRGHV | 1.1 | 7 |  |  |  |  |  | 4 |
| **Total sequences** |  |  | **23** | **33** | **32** | **27** | **27** | **26** |
